# Searching and Evaluating Publications and Preprints Using Europe PMC

**DOI:** 10.1002/cpz1.694

**Published:** 2023-03-01

**Authors:** Summer Rosonovski, Maria Levchenko, Michele Ide-Smith, Lynne Faulk, Melissa Harrison, Johanna McEntyre

**Affiliations:** 1 Literature services, EMBL-EBI, Wellcome Trust Genome Campus, Cambridge, United Kingdom

**Keywords:** Europe PMC, information retrieval, literature search, preprint, PubMed, evaluating publications

## Abstract

In the field of life sciences there is a growing need for literature analysis tools that help scientists tackle information overload. Europe PubMed Central (Europe PMC), a partner of PubMed Central (PMC; National Library of Medicine, 2022), is an open access database of over 41 million life science publications and preprints, enriched with supporting data, reviews, protocols, and other relevant resources. Europe PMC is a trusted repository of choice for many life science funders (Europe PMC, 2022a), offering a suite of innovative search tools that allow users to search and evaluate the literature, including finding highly cited articles, preprints with community peer reviews, or papers referencing a proteomics dataset in the figure legend. In addition, Europe PMC utilizes textmining to help researchers identify key terms and find data and evidence in the literature. First-time users often do not utilize the wealth of tools Europe PMC offers and can feel overwhelmed about how to perform the most effective search. This protocol, describing how to search and evaluate publications and preprints using Europe PMC, demonstrates how to carry out more efficient and effective literature searches using the tools provided by Europe PMC. This includes discovering the latest findings on a research topic, following research from a specific author, journal, or preprint server, exploring literature on a new method, expanding your reading list with relevant articles, as well as accessing and evaluating publications and preprints of interest.

**Basic Protocol 1:** Finding articles and preprints on a topic of interest

**Basic Protocol 2:** Accessing an article

**Basic Protocol 3:** Browsing the article

**Basic Protocol 4:** Evaluating the article

**Basic Protocol 5:** Refining search results

**Basic Protocol 6:** Finding research by author

**Basic Protocol 7:** Finding a specific article

**Basic Protocol 8:** Finding information about a methodology

**Basic Protocol 9:** Finding evidence of biological interactions, relations, and modifications

**Basic Protocol 10:** Finding data behind a publication

**Basic Protocol 11:** Expanding a reading list and building a bibliography

**Basic Protocol 12:** Staying on top of the current literature

## Introduction

For researchers, staying current with the literature in their field is a crucial task. However, scientists are facing many challenges when it comes to staying ahead of the information overload. For instance, finding evidence of biological events, interactions, or relations is an important part of literature analysis, but it often relies on manual literature searches and curation, making it difficult to scale and requiring a significant time commitment. The explosion in the amount of data reported in the literature due to technological advancements in high throughput technologies (Regnard, 2021), makes it difficult to identify references to relevant datasets from the large corpus of publications. Another example is the challenge of tracking preprints. During the COVID-19 pandemic, use of preprints for reporting new findings accelerated dramatically; however, preprints remain scattered across many platforms and are difficult to search alongside traditional peer reviewed publications. In addition, as preprints continue to gain popularity, new ways of effectively evaluating the literature beyond the traditional journal-organized peer review is required. This publication describes how to use the suite of tools provided by Europe PubMed Central (Europe PMC) for analyzing and evaluating life science literature. Europe PMC is a global, free database of life science publications and preprints, all freely accessible via the website, RESTful application programming interface (API), and bulk download (Europe PMC, 2022c). As a part of the PubMed Central (PMC; National Library of Medicine, 2022) and International archive network (NIH/NLM, 2022), Europe PMCprovides access to over 8 million full-text articles and over 40 million abstracts from PubMed/MEDLINE (NIH/NLM, 2022), PubMed Central (National Library ofMedicine, 2022), Agricola (AGRICOLA, 2022), various preprint servers, and other trusted sources. Preprints can be searched alongside journal articles, and are linked to scientific commentary, peer reviews and recommendations, as well as traditional and alternative metrics. Publications and preprints in Europe PMC are linked to open data from over 40 life science databases, supporting reuse and reproducibility. Europe PMC uses text mining and machine learning techniques to annotate publications with relevant biological terms and concepts, from chemicals and cell lines to gene mutations, target-disease associations, and protein interactions, helping to extract evidence from the literature. Not all Europe PMC tools are available on all articles due to licensing constraints, for example, figure previews and annotations cannot be displayed unless the article is published under a CC-BY license (Creative Commons, 2019).

In this article, we provide detailed protocols describing how to effectively search and evaluate publications and preprints using Europe PMC. Basic Protocol 1 walks the user through the steps to find articles and preprints on a particular topic of interest using the Europe PMC main search bar. It covers accessing the Europe PMC home page, doing a basic keyword literature search, and an exact phrase search. Basic Protocol 2 describes how to access the article full text and walks the user through the article layout. Basic Protocol 3 explains how to browse through the specific article sections, explore figures, and quickly scan the article for biological concepts of interest. Basic Protocol 4 describes how a user can access version history, peer review status and materials, as well as impact metrics to help with article or preprint evaluation. Basic Protocol 5 explains how users can assess the relevance of search results and refine their search strategy. Basic Protocol 6 describes how to search for records by a particular author, including search by Open Researcher and Contributor IDentifier (ORCID; ORCID, 2022). Basic Protocol 7 walks the user through the steps to find a specific article using the main search bar and Advanced search. Basic Protocol 8 explores how a user can limit their search to a particular article to find information about a methodology, explore methods, reagents, and biologicals used in the article, as well as access additional protocols. Basic Protocol 9 shows the user how to find evidence in the literature, including biological interactions, relations, and modifications, using literature analysis tools developed by Europe PMC. Basic Protocol 10 highlights how to access the data behind the publication or find literature citing particular datasets. Basic Protocol 11 describes how users can expand their reading list with similar or citing articles and build a bibliography using tools provided by Europe PMC. Basic Protocol 12 describes how to set up alerts for new publications on a particular topic to stay on top of the current literature.

## Finding Articles and Preprints on a Topic of Interest (Basic Protocol 1)

In this protocol, we explain how to find articles and preprints on a topic of interest by searching the Europe PMC database using the main search bar. Alternatively, users can search for articles on a particular topic using Europe PMC Advanced search. This protocol describes how to find relevant articles and preprints using a keyword search or exact phrase search, and how to navigate the search results page.

### Necessary Resources

#### Hardware

Device capable of supporting a Web browser and an Internet connection

#### Software

Up-to-date Web browser (e.g., Firefox, Apple Safari, Google Chrome)

**Figure 1:**
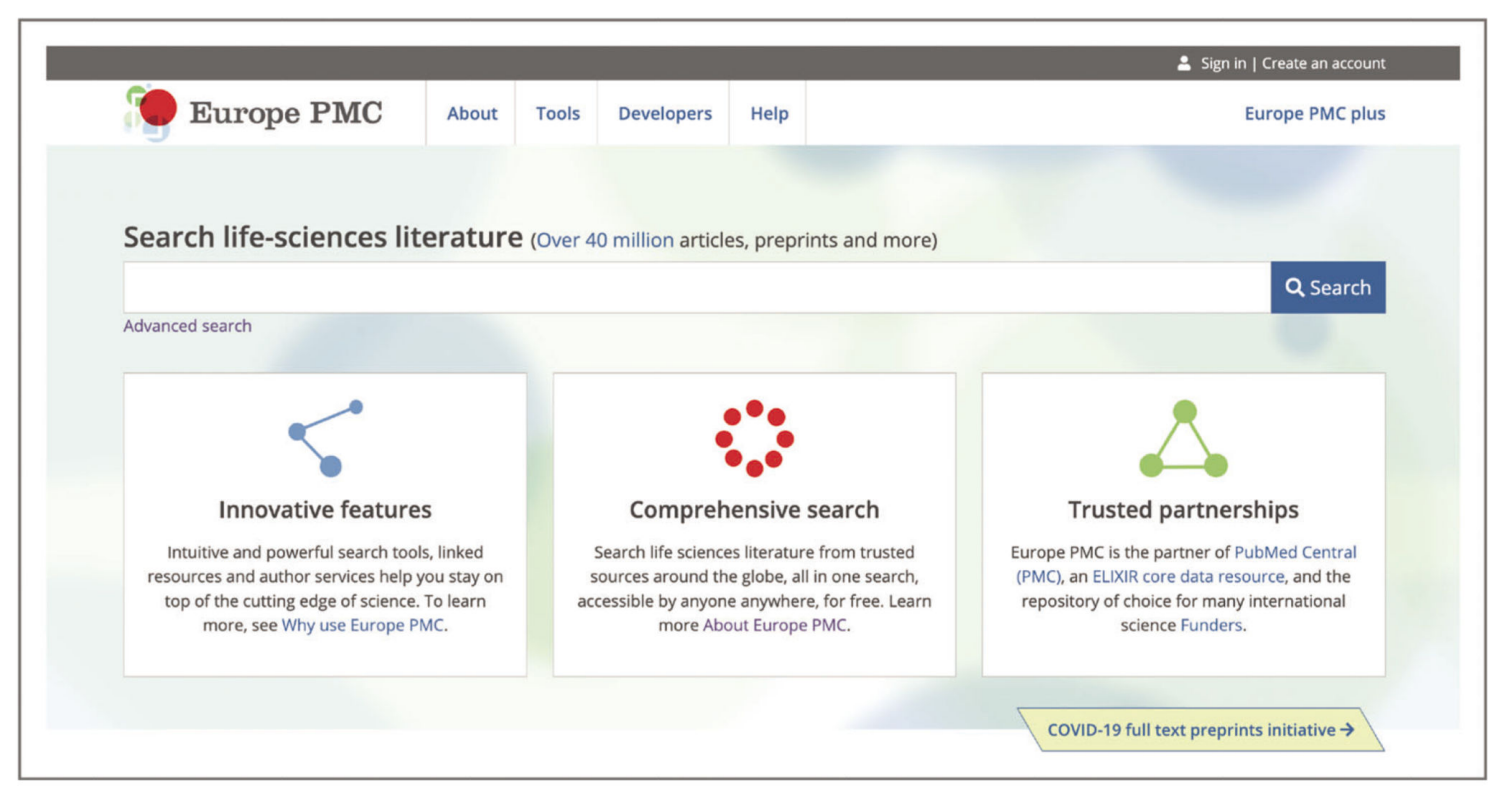
The Europe PMC homepage with the main search bar.

**Figure 2:**
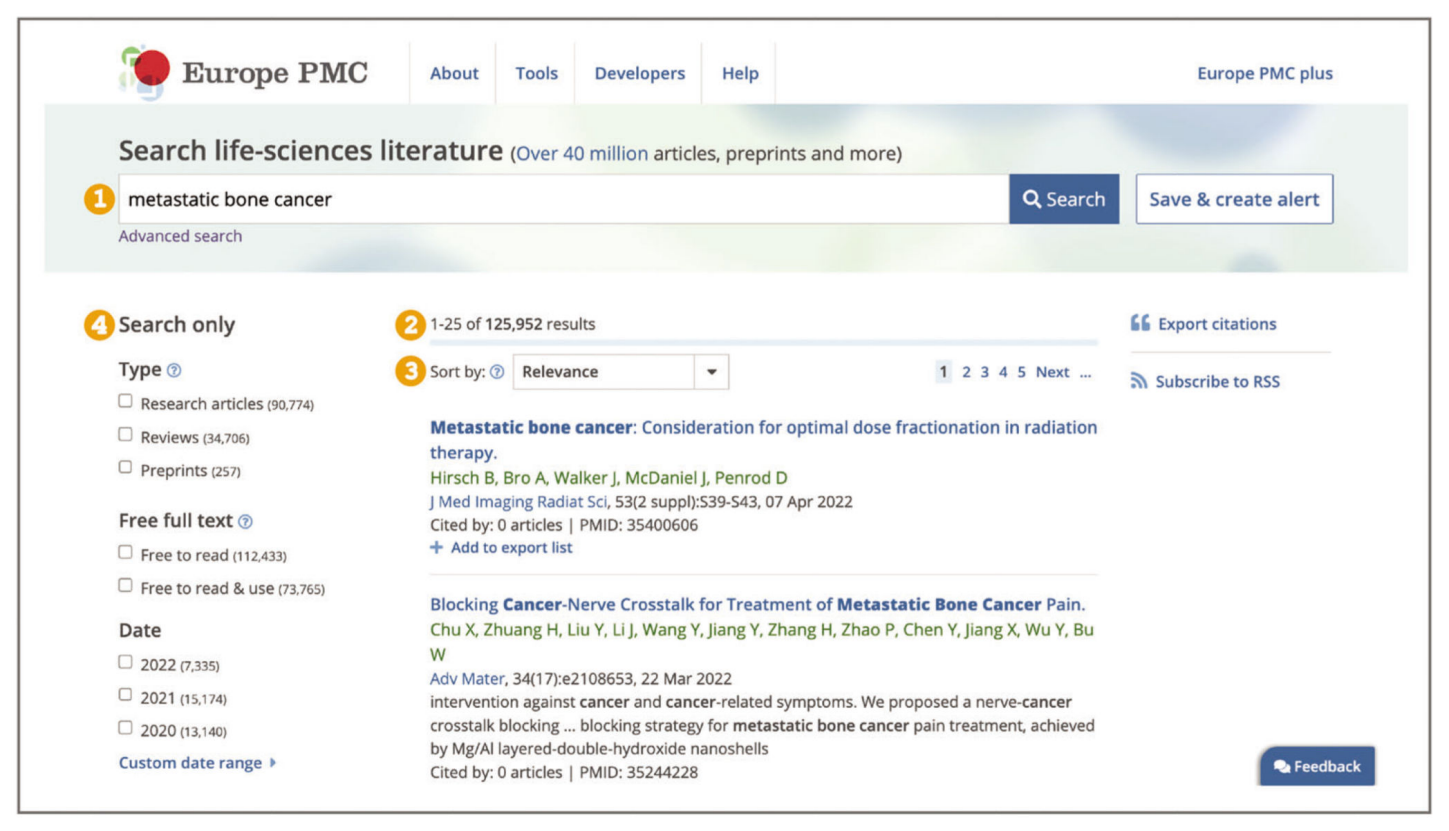
Search results for the terms ***metastatic bone cancer*** entered into the Europe PMC main search bar. (1) Terms ***metastatic bone cancer*** in the Europe PMC main search bar. (2) The number of results. (3) Sort order. (4) Search filters. URL used for the screenshot: https://europepmc.org/search?query=metastatic%20bone%20cancer

**Figure 3:**
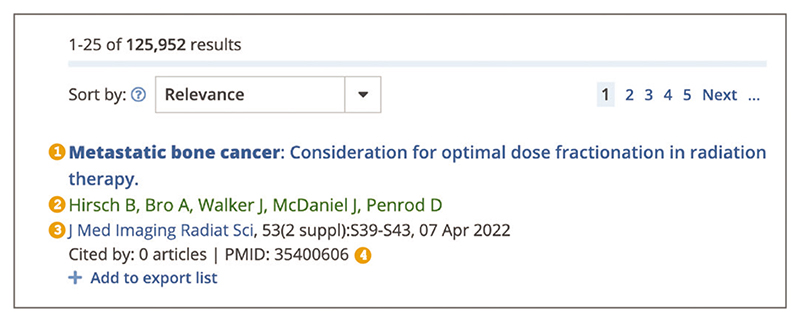
Zoomed in view of the first search result for ***metastatic bone cancer*** search. (1) Title of the record. (2) Authors. (3) Publication details, including journal or preprint server, volume, issue, pagination, and publication date. (4) Publication orpreprint identifiers. URL used for the screenshot: https://europepmc.org/search?query=metastatic%20bone%20cancer

1. Open the Europe PMC homepage http://europepmc.org in a browser of your choice (Fig. 1).
2. To perform a simple search, type a keyword, or a string of terms, such as **metastatic *bone cancer***, into the main search bar and select the ‘Search’ button.
  A search for a term or a string of terms, such as **metastatic bone cancer**, will bring up publications containing **metastatic** and **bone** and **cancer**, but the words may appear anywhere in the text and are not necessarily in close proximity to each other.
  Search coverage: The Europe PMC search will return all records, including journal articles and preprints, where the search term or multiple terms are found in the abstracts, metadata, or full text, where available. Here metadata includes information about the record, such as authors, title, journal, or keywords. Note that the search excludes references. See Basic Protocol 5 for steps to limit search to a specific article section.
3. It is also possible to search for an exact phrase match using quotation marks, for example ***“tRNA function”***. Exact search retrieves publications where the entire phrase is present in the same sequence as specified.
  A key difference between a term search, such as **tRNA function**, and an exact phrase search, such as **“tRNA function”**, is that the term search will bring up publications containing both **tRNA** and **function**, but the words may appear anywhere in the text and are not necessarily in close proximity to each other. For example, both articles titled “tRNA Function and Dysregulation in Cancer” and “Function and Therapeutic Implications of tRNA Derived Small RNAs” will be picked up by **tRNA function** keyword search, but only the first article will be retrieved by the **“tRNA function”** exact phrase search.
4. To retrieve a broader number of results, search terms are modified by the search engine to be case insensitive, special characters are replaced, and stop words and punctuation are removed.
  Searches in the main search bar are case insensitive. For example, when searching for **MYC**, results containing **myc** will also be found. Searches filter out stop words, such as “a”, “on”, or “is” (Solr, 2022). For example, if **downstream of rac1** is typed into the main search bar, the search performed will be **downstream rac1**. Stop words remain for exact phrase searches, such as **“downstream of rac1”**. Punctuation and special characters are removed from keyword searches with few exceptions (Solr, 2022).
5. Search results are displayed on the search results page (Fig. 2). The results page shows the total number of results, the list of the first 25 results, as well as the navigation to further results pages. The results page also provides options to change sort order, export results, and refine search using filters (described in Basic Protocol 2).
6. For each record on the results page (Fig. 3), the title, authors, journal or preprint server, volume, issue, pagination, publication date, and identifiers, e.g., PubMed identifier (PMID), PubMed Central identifier (PMCID), unique preprint identifier generated by Europe PMC (PPR), are displayed where available.

## Accessing an Article (Basic Protocol 2)

This protocol walks the user through the steps to access an article or preprint in Europe PMC, including free full text, where available.

### Necessary Resources

#### Hardware

Device capable of supporting a Web browser and an Internet connection

#### Software

Up-to-date Web browser (e.g., Firefox, Apple Safari, Google Chrome)

**Figure 4:**
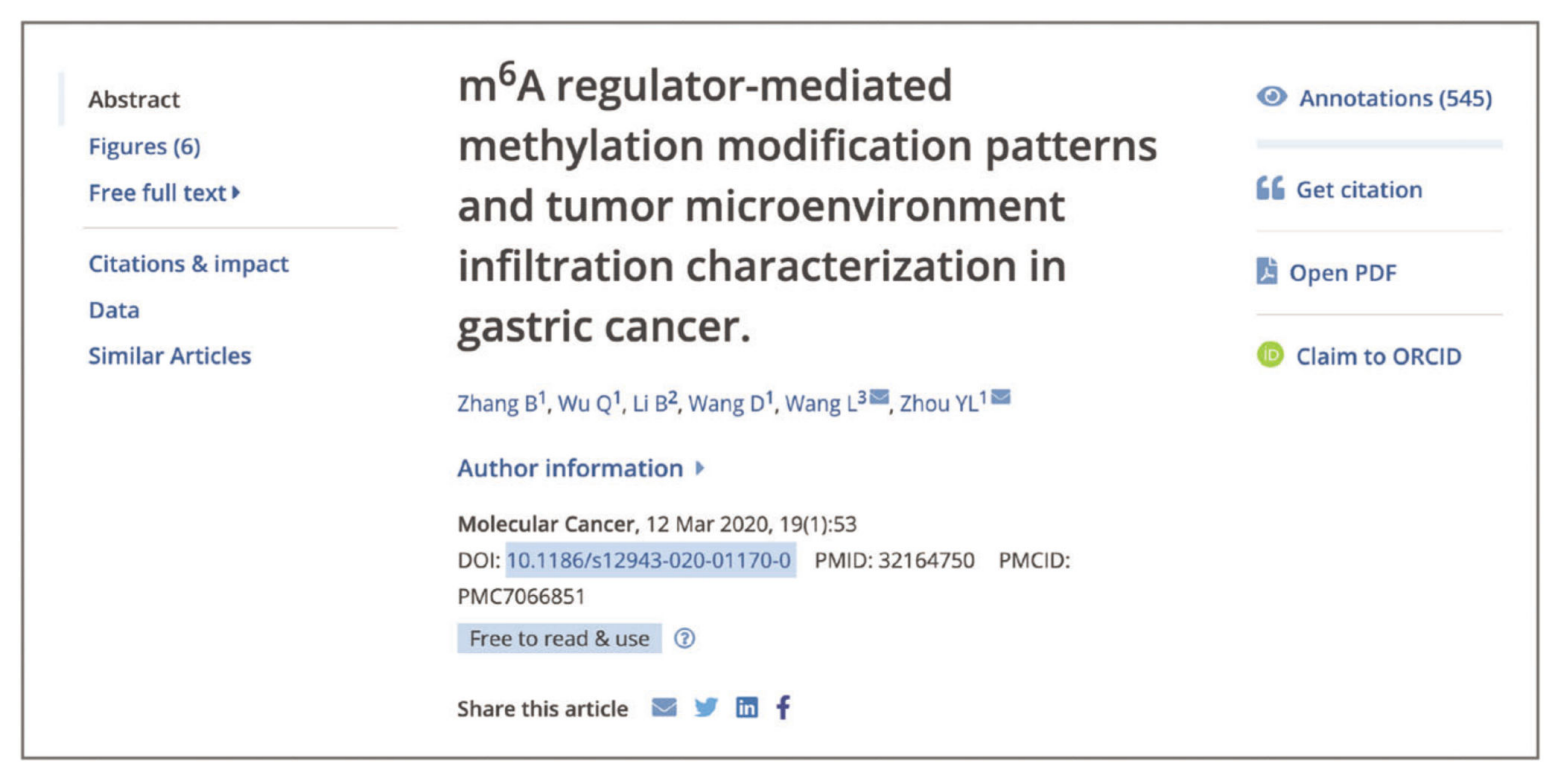
An article page in Europe PMC. Article text, authors, and citation information are displayed in the center. The navigation bar is displayed on the left-hand side with headings allowing you to jump to specific sections, such as Abstract, Figures, Free full text, and more. The tools bar is displayed on the right, with options to access Annotations (Basic Protocol 3, steps 3-4), Get citation (Basic Protocol 11, step 2), Open PDF (Basic Protocol 2, step 4), or Claim to ORCID. Article URL used for screenshot: https://europepmc.org/article/MED/32164750

**Figure 5:**
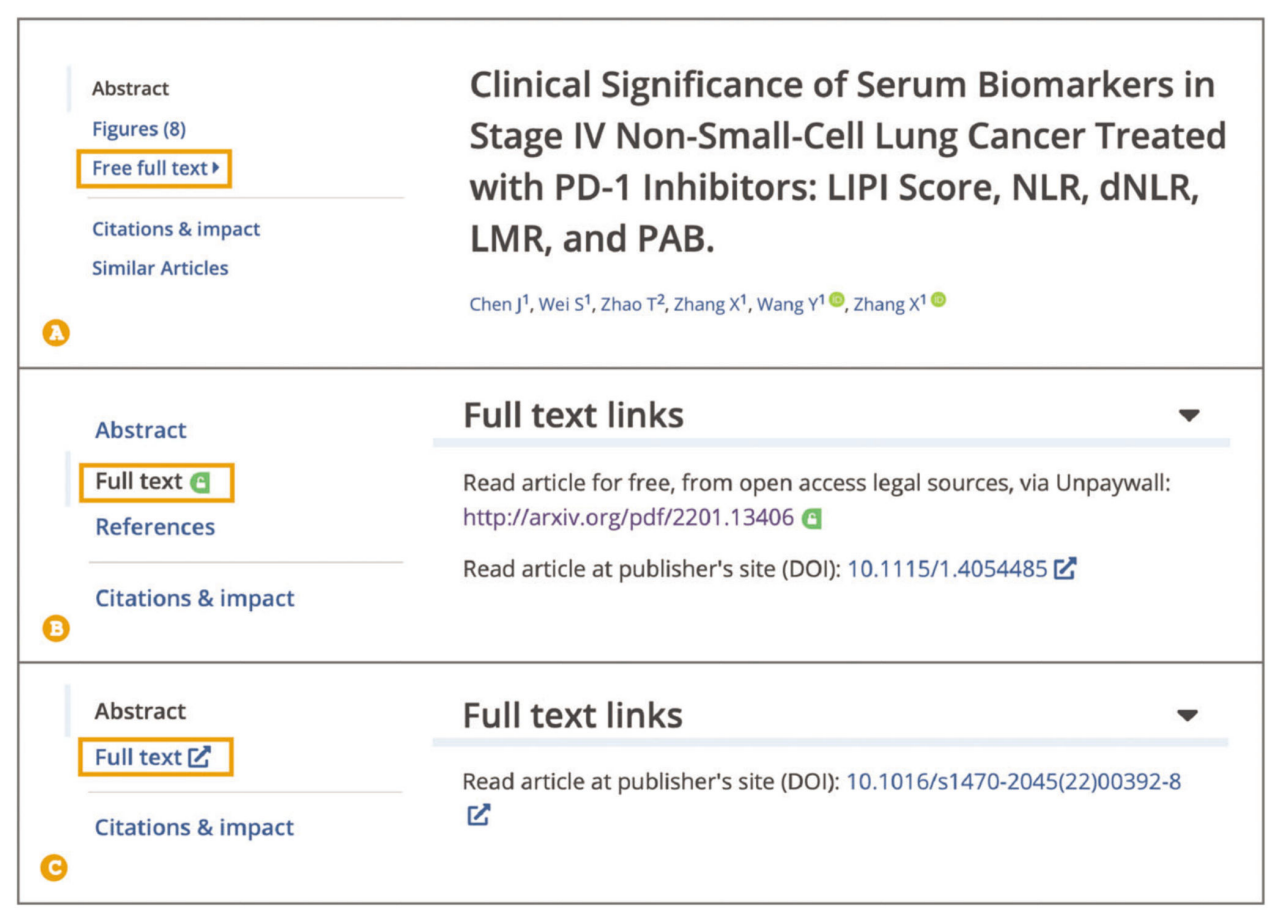
Free full-text section of the article page in Europe PMC. (**A**) Free full text link in the left-hand navigation panel for a publication with free full text in Europe PMC. (**B**) Full text link with the padlock icon on the green background indicates an external link to the free legal copy of the publication full text available via Unpaywall. (**C**) Full text link with an external link icon indicates that the full text can be accessed on the publisher’s website. Article URLs used for the screen-shots: https://europepmc.org/article/PMC/PMC9357262(A),https://europepmc.org/article/MED/35510823 (B), https://europepmc.org/article/MED/35934010 (C).

1. Users can access further information about the article or preprint, including abstract and full text, where available, by navigating to the article page via the result title hyperlink from the search results page.
2. All the content related to the article is provided on the same page, including data, reviews, citations, and other useful resources (Fig. 4).
3. To access the full text of the publication, use the navigation bar on the left-hand side and select ‘Free full text’ or ‘Full text’. If the free full text is available in Europe PMC, a ‘Free full text’ link is provided on the article page in the navigation bar on the left (Fig. 5A). If a free, legal copy of the full text is found on an external website, a ‘Full text’ link is shown along with a padlock icon on green background (Fig. 5B). If the full text is only available at the publisher website, a ‘Full text’ link is shown along with an external link icon (Fig. 5C).
  As of September 2022, full text is freely available in Europe PMC for over 8.1 million records. Europe PMC provides access to additional free, legal copies of full-text articles on external websites via Unpaywall, an open database of free scholarly articles (Unpaywall, 2022).
4. Where full text is available in Europe PMC, you can access a PDF version of the fulltext article by selecting the ‘Open PDF’ option via the Tools bar on the right-hand side (Fig. 4).

## Browsing the Article (Basic Protocol 3)

This protocol describes how to browse through the article sections, explore figures, and scan the article for genes, diseases, organisms, and other concepts of interest.

### Necessary Resources

#### Hardware

Device capable of supporting a Web browser and an Internet connection

#### Software

Up-to-date Web browser (e.g., Firefox, Apple Safari, Google Chrome)

**Figure 6:**
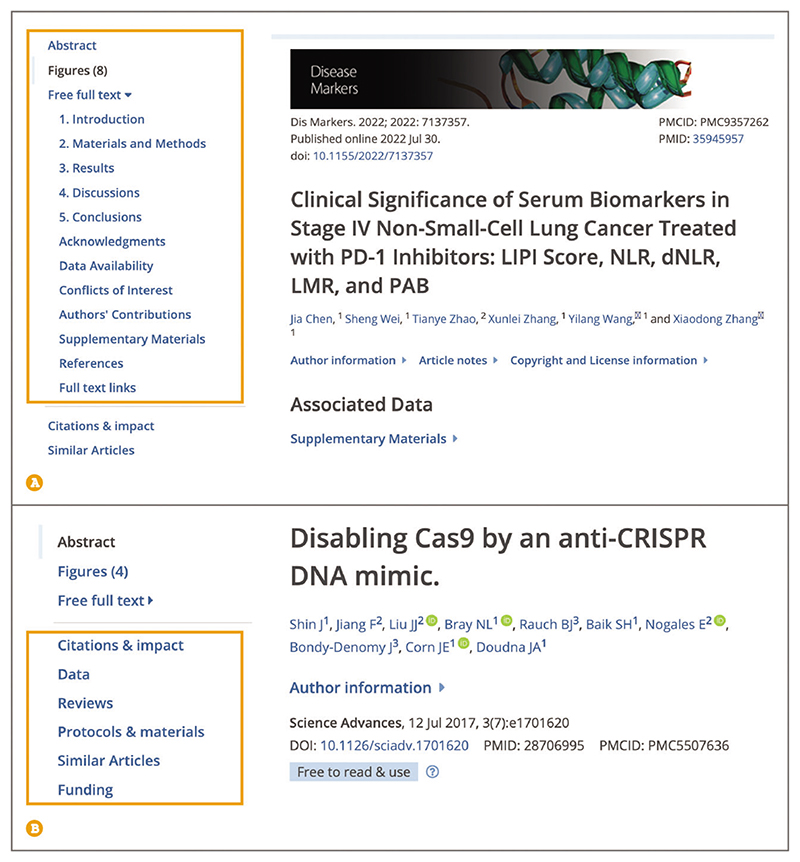
The navigation panel on the left-hand side of the article page contains links to the Abstract, Figures, Free full text, and Europe PMC generated sections such as Citations & impact, and Similar Articles. (**A**) Free full text further expands to include the headings provided in the article, in this example this includes Introduction, Materials and Methods, Results, Discussions, Conclusions, Acknowledgments, Data Availability, Conflicts of Interest, Authors’ Contributions, Supple-mentary Materials, and References. (**B**) An article page showing Europe PMC generated sections, including Citations & impact, Data, Reviews, Protocols & materials, Similar Articles, and Funding. Article URLs used for screenshots: https://europepmc.org/article/PMC/PMC9357262 (A), https://europepmc.org/article/MED/28706995 (B).

**Figure 7:**
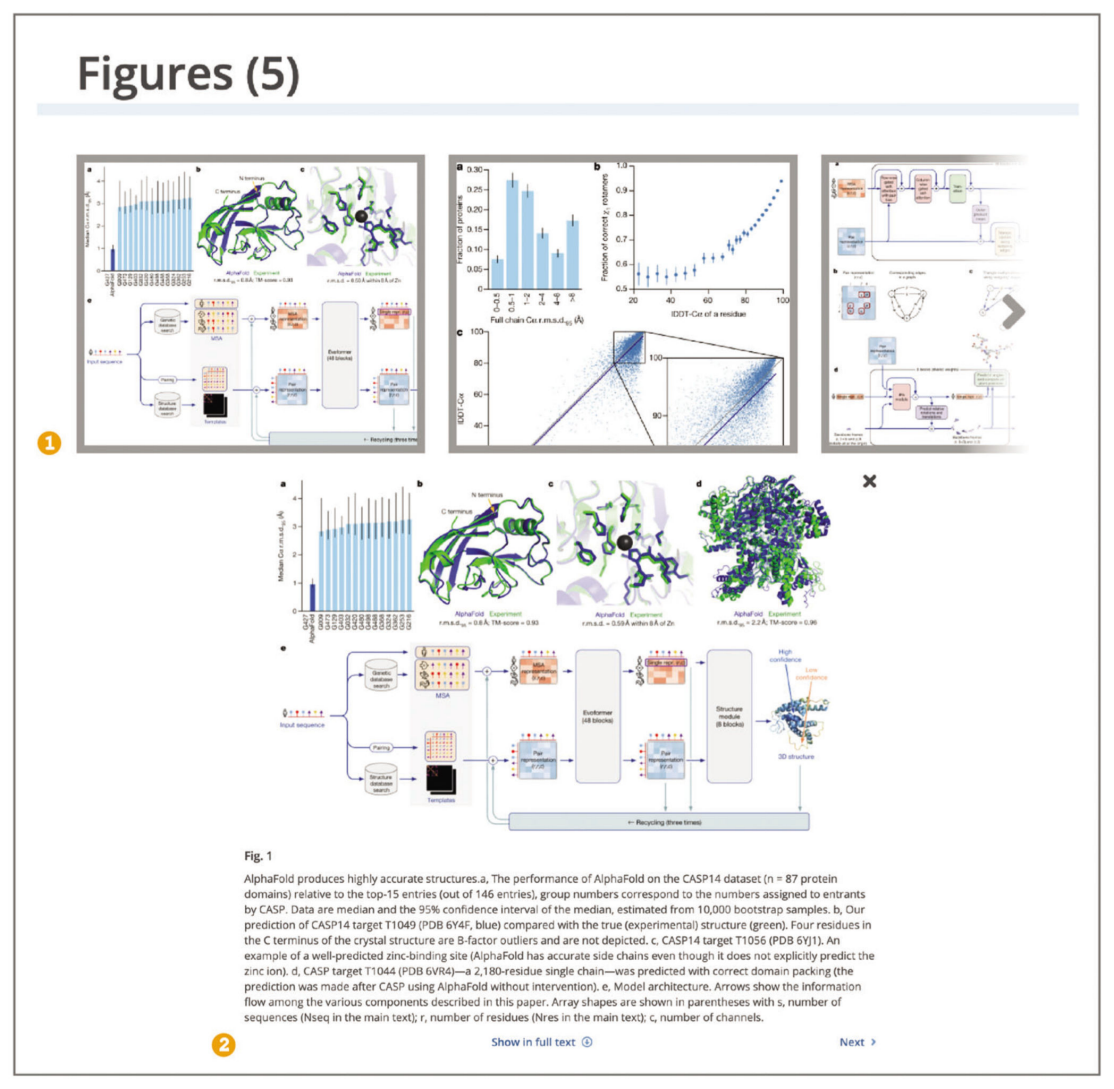
Figures from the publication are shown as thumbnails on the article page (1). The expanded view for Figure 1 includes the figure legend, the Next navigation arrow, and Show in full text option (2).ArticleURL used for the screenshot:https://europepmc.org/article/MED/34265844

**Figure 8:**
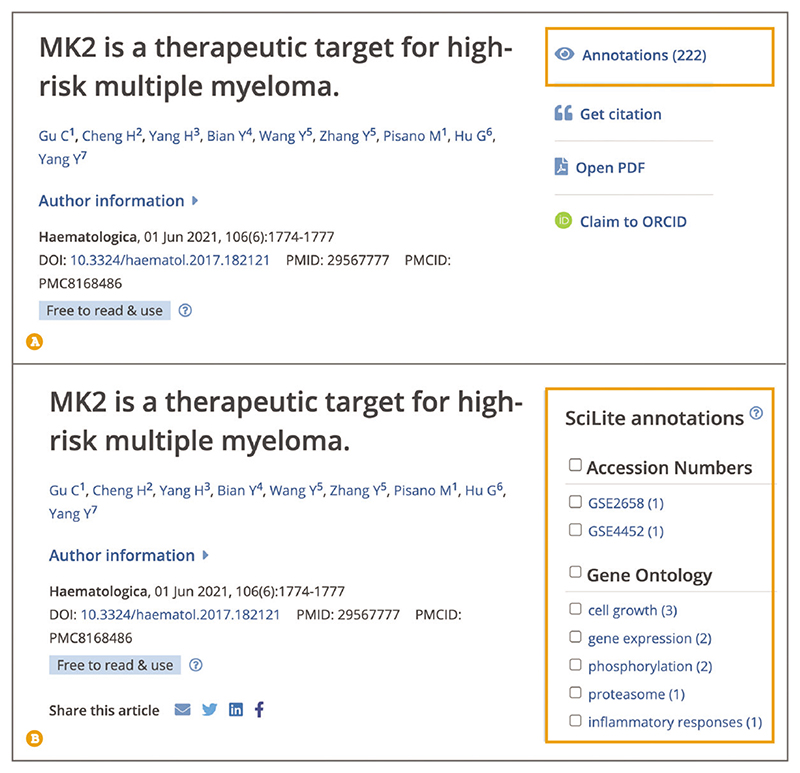
(**A**) An article page with the Annotations option highlighted in the toolbar on the righthand side. (**B**) An open Annotations panel that lists different biological concepts organized into broad categories. Individual terms below each category are listed along with the number of occurrences in the abstract and full text. Article URL used for the screenshot: https://europepmc.org/article/MED/29567777

**Figure 9:**
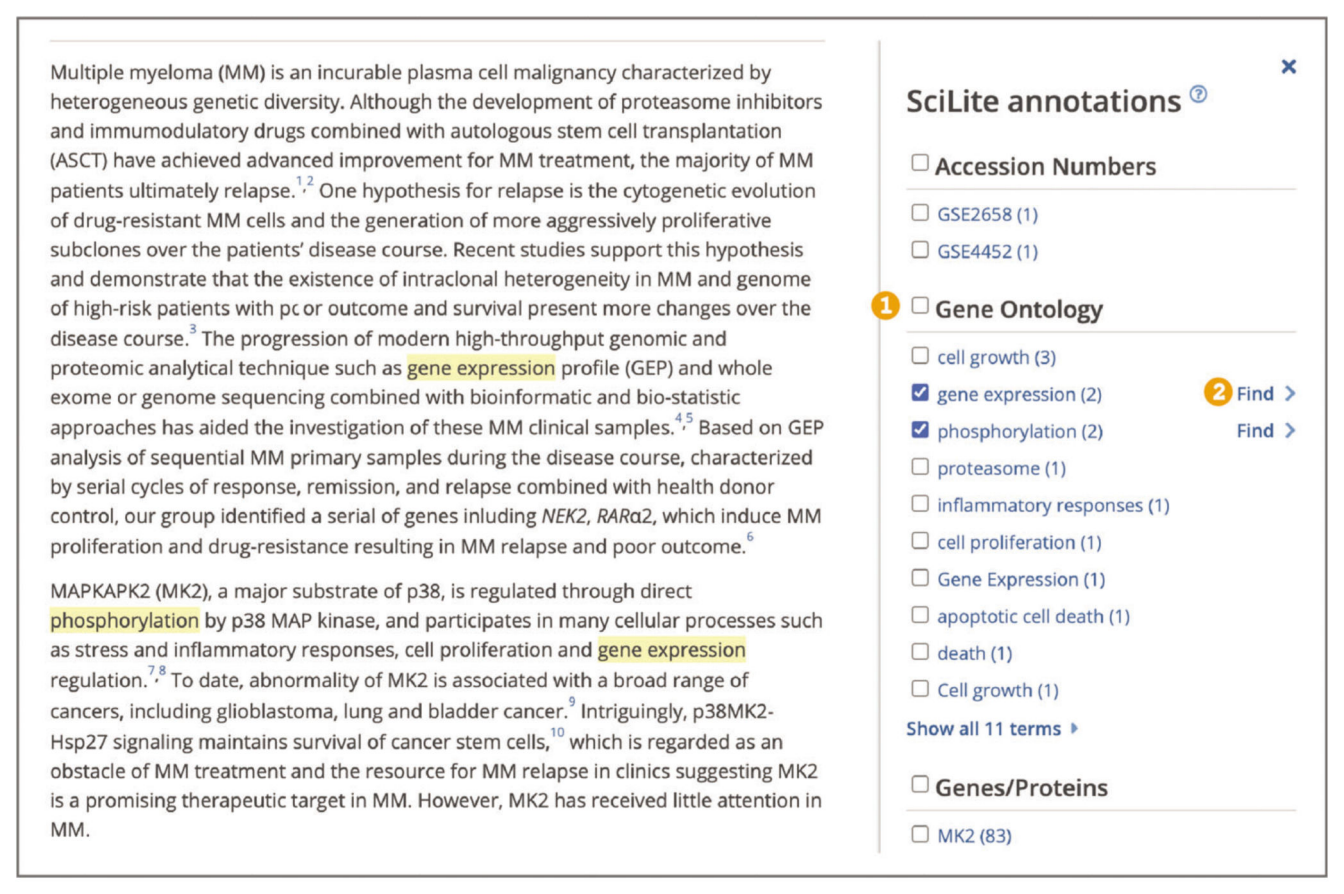
Annotations panel open with gene expression and phosphorylation terms selected from the Gene Ontology category (1). The selected terms are highlighted throughout the text. Find link on the right-hand side of the selected concept (2). Article URL used for the screenshot: https://europepmc.org/article/MED/29567777

1. When browsing the full text of a publication, use the navigation bar to jump straight to the section of interest (Fig. 6). The publication sections, such as ‘Introduction’, ‘Methods’, ‘Results’, or ‘Discussion’, can be accessed after expanding the Free full text section.
  Information on article sections is provided to Europe PMC by the publisher, and the list of sections available for each article will differ.
  Sections listed below the Full text section (Fig. 6B), such as ‘Citations & impact’, ‘Data’, ‘Protocols & materials’, ‘Reviews’, ‘Lay summaries’, ‘Similar Articles’, and ‘Funding’, are generated by Europe PMC and are not a part of the publication text.
2. To view publication figures at a glance, go to the ‘Figures’ section from the navigation bar or scroll below the abstract (Fig. 7). Use arrows to navigate between figure thumbnails. To get an expanded view, including the figure legend, select the individual figure thumbnail. To view a figure of interest in context, open the ‘Show in full text’ link.
  Figure preview carousel can only be shown under the abstract if the full text is available in Europe PMC and the article has a license that allows reuse and modification. For articles where the full text is available on Europe PMC, the figures will be embedded throughout the text and for articles where the full text is not available, contact the author for a copy of the full-text publication.
3. To quickly scan the article for relevant concepts, such as organisms, chemicals, diseases, or genes and proteins, open the ‘Annotations’ panel from the tools bar on the right-hand side (Fig. 8).
  Annotations are biological terms, such as diseases, chemicals, or protein interactions, identified using text-mining algorithms from a variety of expert text-mining groups (Europe PMC, 2022b). As of September 2022, Europe PMC lists over 40 different annotation types.
4. To highlight relevant annotations in the text, select the term or several terms of interest in the ‘Annotations’ panel. You can navigate through annotated concepts by using the ‘Find’ option, which will locate the next mention of the concept in the article text (Fig. 9).
  Annotations can only be highlighted on abstracts and full-text articles with a license that allows reuse and modification.

## Evaluating the Article (Basic Protocol 4)

This protocol describes how to access version history, peer review status and materials, as well as citations and other impact metrics to evaluate articles and preprints of interest.

### Necessary Resources

#### Hardware

Device capable of supporting a Web browser and an Internet connection

#### Software

Up-to-date Web browser (e.g., Firefox, Apple Safari, Google Chrome)

**Figure 10:**
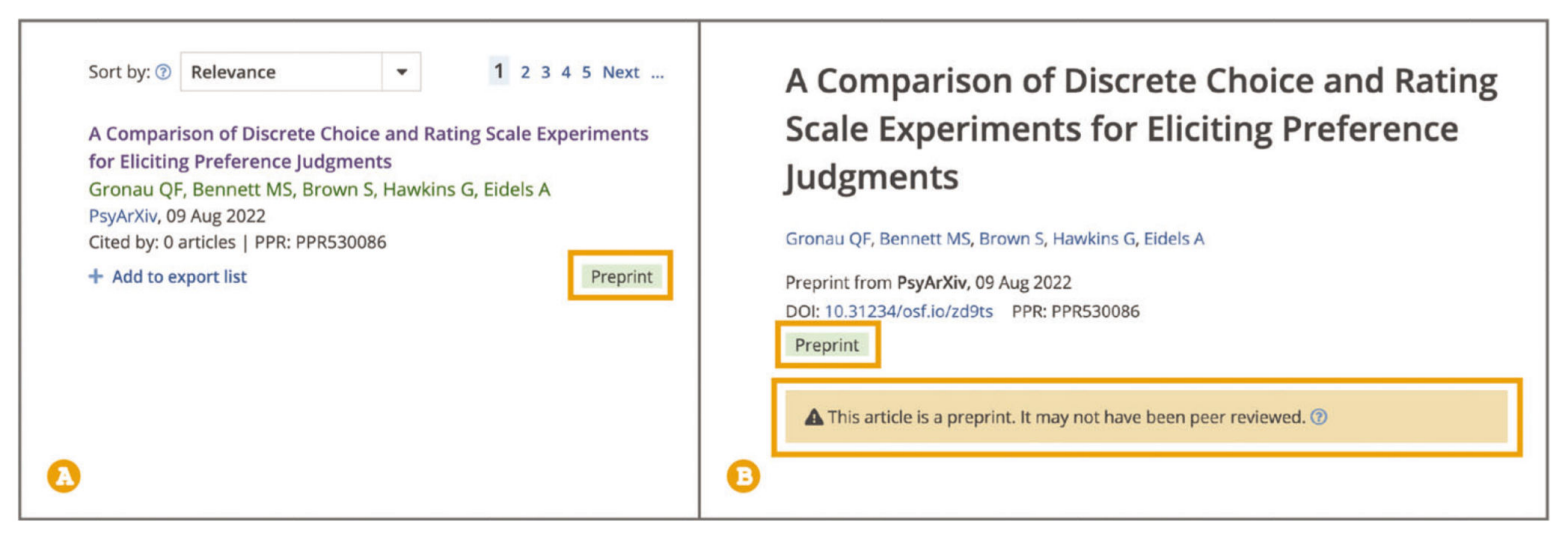
(**A**) Search result with a green Preprint label. (**B**) Preprint page showing the Preprint label and an orange notification box stating that the article is a preprint and may not yet have been peer reviewed. Preprint URL used for the screenshot: https://europepmc.org/article/PPR/PPR530086

**Figure 11:**
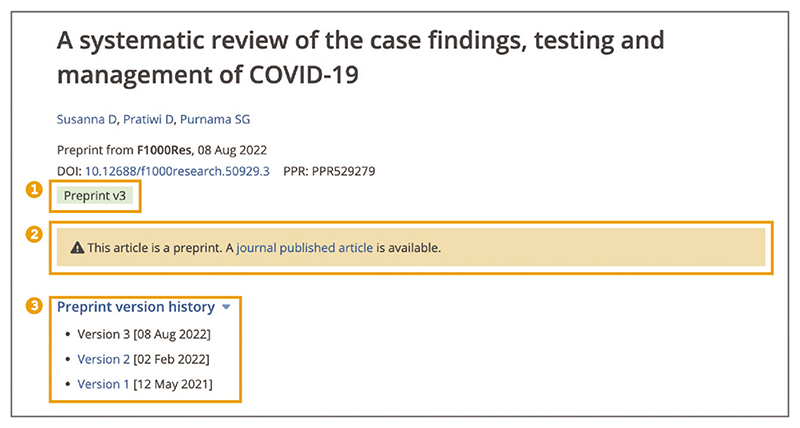
Preprint page. A green Preprint label indicates version 3 (1), orange notification box with a link to the journal published article (2), and Preprint version history (3). Preprint URL used for the screenshot: https://europepmc.org/article/PPR/PPR529279

**Figure 12:**
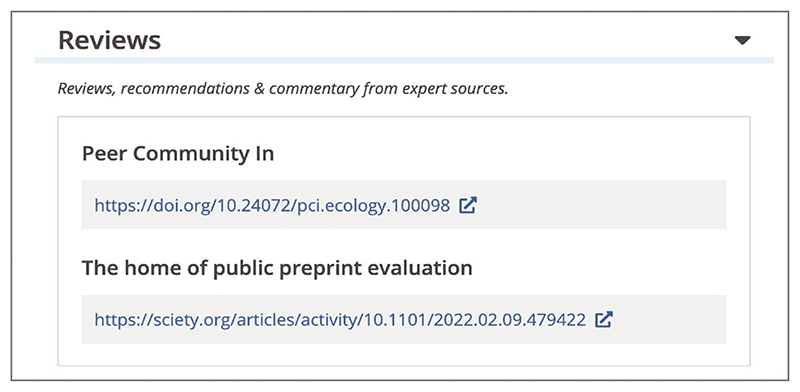
Article page Reviews section with links to reviews available via Peer Community In (Peer Community In, 2022) and Sciety (Sciety, 2022). Preprint URL used for the screenshot: https://europepmc.org/article/PPR/PPR452155

**Figure 13:**
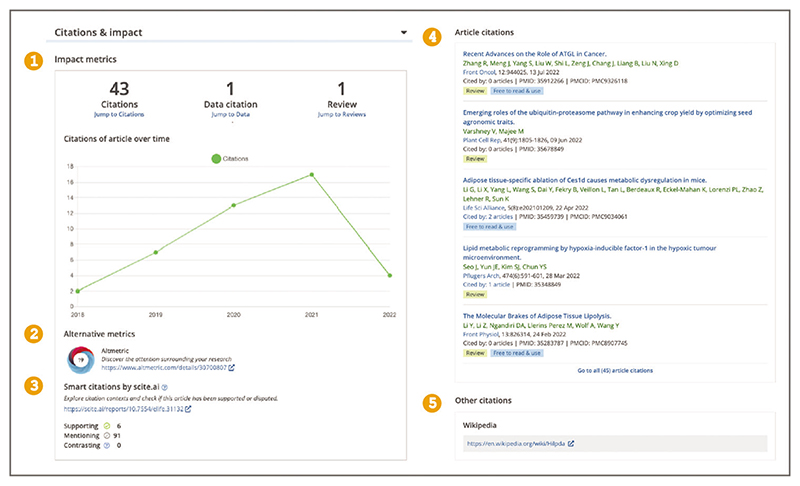
Article page Citation & impact section of an article in Europe PMC. This section displays a yearly citation graph (1), alternative metrics provided by Altmetric (2), Smart citations by scite.ai (3), and other citations, for example a list of citing articles (4), and citations from Wikipedia pages (5). Article URL used for the screenshot: https://europepmc.org/article/MED/29256392

1. To distinguish preprints from peer-reviewed articles in Europe PMC, search results look for a green Preprint label (Fig. 10A). Preprint records also display an orange notification box on the preprint page that warns the reader that the preprint may not have been peer reviewed (Fig. 10B).
  Preprints are complete scientific manuscripts uploaded by the authors onto a public server. A preprint is posted without peer review but may acquire feedback or reviews at a later stage or be eventually published in a peer-reviewed journal. Europe PMC allows you to search preprints across more than twenty different preprint platforms, including bioRxiv (bioRxiv, 2022), medRxiv (medRxiv, 2022), ResearchSquare (Research Square, 2022), and many others.
2. To access the peer-reviewed journal version of the preprint (if available) open the ‘journal published article’ link in the orange notification box. You can access other preprint versions from the ‘Preprint version history’ drop down menu. The current preprint version is indicated in the green Preprint label (Fig. 11).
  Europe PMC retains preprints and versions even if a peer-reviewed journal version is published. This ensures preprint citations can be linked and found. It is also critical to retain versions in support of transparency of sharing of scientific research.
  For preprints to be linked to the peer-reviewed journal article they both must be included in Europe PMC. A link is established based on the title and first author match. A link might be missed due to title and/or first author changes from preprint to published article. Preprint version history is the documentation of the initial preprint submission (version 1) and any updated versions that have been released, e.g., version 2, version 3, and so on. They are all crosslinked in Europe PMC to ensure you can access the most up-to-date version of the research. For some preprint servers, such as bioRxiv (bioRxiv, 2022) or medRxiv (medRxiv, 2022), preprint version history is unavailable due to versioning practices established by the server. In these cases, only the latest preprint version will be available in Europe PMC.
3. To view preprint and journal article peer reviews, recommendations, and scientific commentaries from services such as Publons (Publons, 2022), Faculty opinions (Faculty Opinions, 2022), preLights (preLights, 2022), Peer Community In (Peer Community In, 2022), PREreview (PREreview, 2022), Sciety (Sciety, 2022), and others, select the ‘Reviews’ section displayed in the left-hand side navigation bar (Fig. 6B). Links to the open peer review materials and recommendations available via these services are listed in the ‘Reviews’ section (see Fig. 12).
4. You can access article citations and alternative metrics by selecting the ‘Citations & impact’ section displayed in the left-hand side navigation bar (Fig. 6B). This section displays the number of citations of the article in each year since publication, alternative metrics provided by Altmetric (Altmetric, 2022), Smart citations by scite.ai (Scite, 2022), or other citations, for example from data resources or Wikipedia pages (Wikipedia, 2022), as well as article recommendations (Basic Protocol 4, see step 3). A list of citing articles is also provided below (Fig. 13).
  The Citations & impact section is generated by Europe PMC for articles with at least one citation from an article included in Europe PMC and thus does not appear for all articles.
  Article citations are available via the open citation network based on the reference lists of the articles in Europe PMC, as well as those made openly available by publishers via Crossref (Crossref, 2022). Alternative metrics from Altmetric complement citation metrics with a donut display that represents mentions on social media, blogs, policy documents, and mainstream media (Altmetric, 2022). Smart citations by scite.ai are citation statements extracted from the full text of the citing article classified as supporting, mentioning, or contrasting the cited claim to provide context to the original citation (Scite, 2022).

## Refining Search Results (Basic Protocol 5)

This protocol describes how to use the sort order, search filters, and Boolean search to refine results and change the way that results are sorted.

### Necessary Resources

#### Hardware

Device capable of supporting a Web browser and an Internet connection

#### Software

Up-to-date Web browser (e.g., Firefox, Apple Safari, Google Chrome)

**Figure 14:**
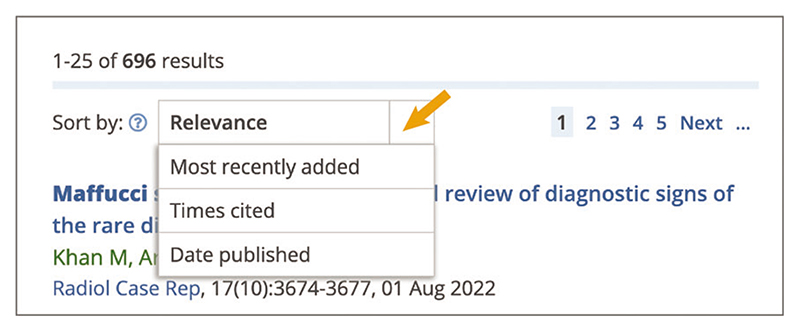
Search results page with an orange arrow pointing to the Sort by drop down box displaying the following sort options: Relevance, Most recently added, Times cited, and Date published.

**Figure 15:**
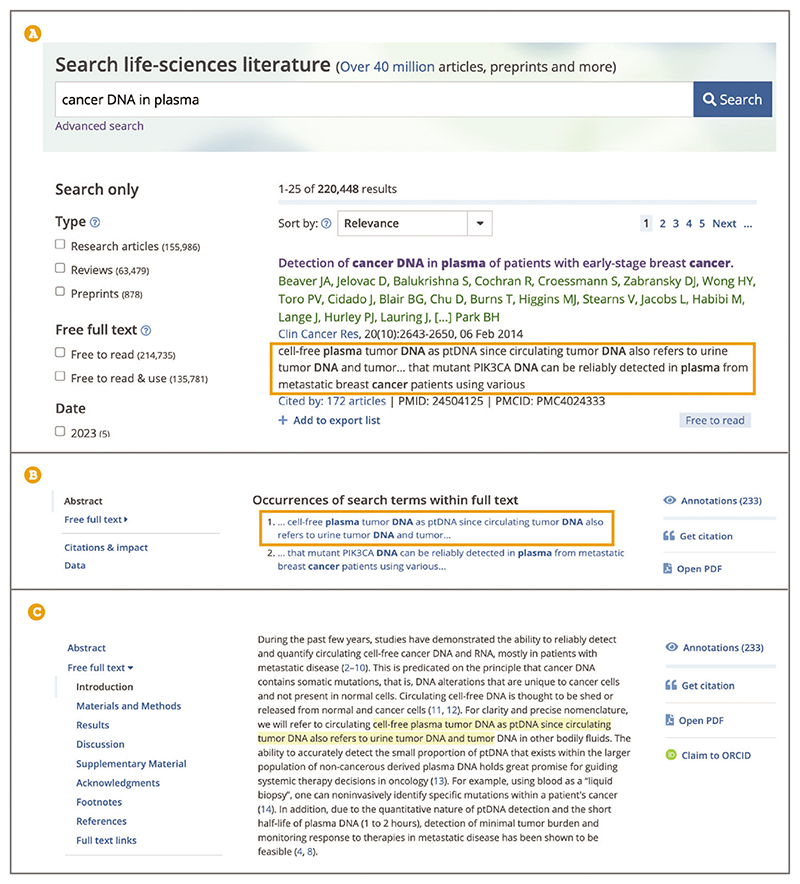
(**A**) The search results page showing a snippet for one of the results highlighted in the orange box. (**B**) The article page showing Occurrences of search terms within the full-text section appearing below the abstract. (**C**) Article page with the snippet shown highlighted in the text for context. Article URL used for the screenshot: https://europepmc.org/article/MED/24504125

**Figure 16:**
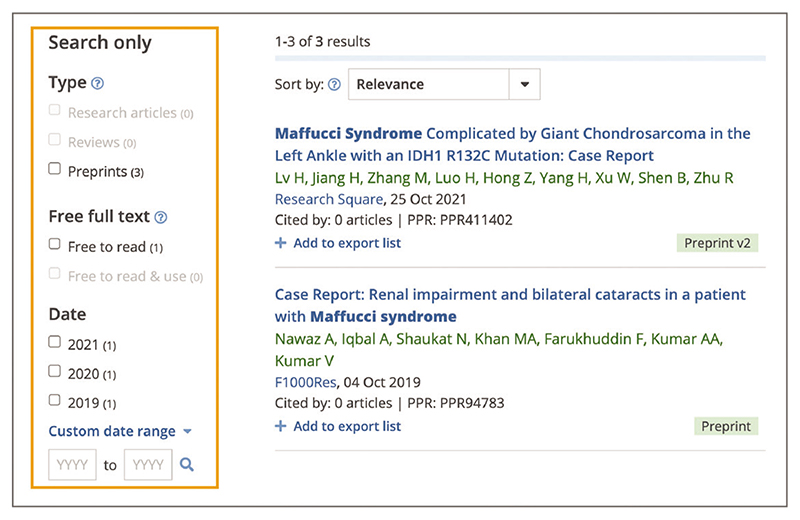
Search filters indicated by an orange box around the Search only section are available from the search results page. URL used for the screenshot: https://europepmc.org/search?query=maffucci%20syndrome%20AND%20%28SRC%3APPR%29&page=1.

**Figure 17:**
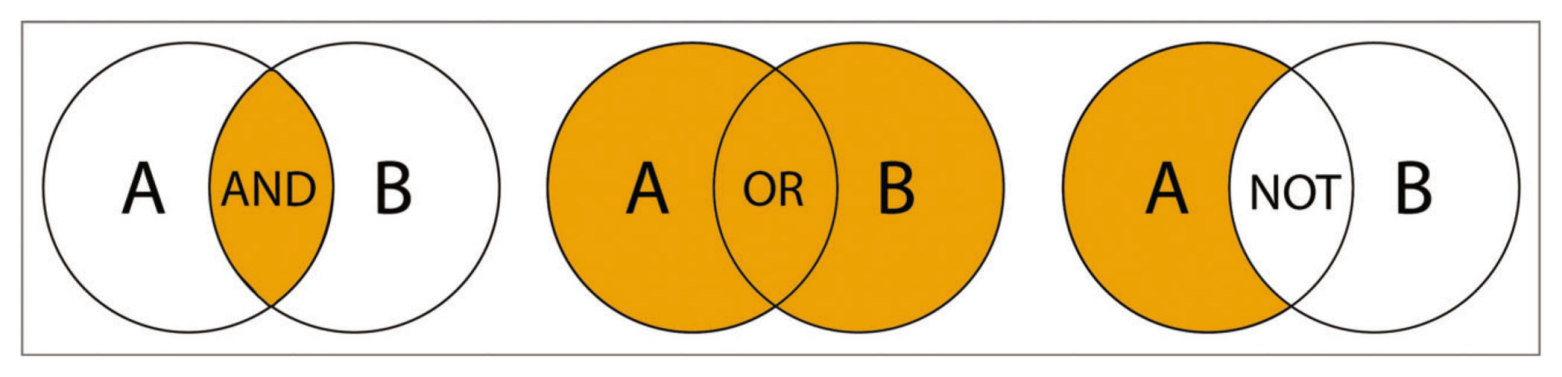
Boolean logic. Figure modified from Icahn School of Medicine at Mount Sinai on PubMed: Combining Terms (Icahn School of Medicine at Mount Sinai, 2022).

1. To help users assess the accuracy of their search, results are displayed in the order of ‘Relevance’. It is also possible to sort your results by ‘Most recently added’ (the date the article was included in Europe PMC), ‘Times cited’, and ‘Date published’. To change the order of results, use the ‘Sort by’ drop-down menu and select the desired option (Fig. 14).
  ‘Relevance’ sorting provides you with the most relevant articles for your search. To do this, Europe PMC gives a higher placement for articles with a combination of features:
  - High search term frequency (the more a search term appears in the article, the higher the article ranks);
  - High number of different search terms (the more of your search terms appear in the article, the higher the article ranks);
  - Rare search terms (rarer search terms appearing in the article makes the article rank higher, for example, amongst results for **maffucci OR syndrome** search, articles containing **maffucci** will rank higher than articles containing **syndrome**, as **maffucci** is a rarer term;
  - Short content (shorter articles containing the keywords will be ranked higher than longer articles, as the statistical probability of the terms appearing in shorter texts is smaller and thus, they are considered to be more relevant);
  - Recent publication date.
2. To further assess the relevance of your search results you can look at the search snippets, sentences from the article that match your keyword(s) search, with keyword(s) appearing in bold (Fig. 15A). Snippets provide useful context for the search, making it possible to judge if it is a negligible mention or an essential reference.
3. To see the snippets in context and find them within the article text, from the search results list select the title of the search result you are interested in, as shown in Figure 15A; this will open the article page. Scroll down below the abstract to ‘Occurrences of search terms within full text’, as shown in Figure 15B, which shows two excerpts from the article that contain your search terms. Select the snippet you want to view. This will take you to where the snippet is located within the full text, Figure 15C.
4. Where necessary, search results can be further refined using the following filters: ‘Type’, ‘Free full text’, and ‘Date’ (Fig. 16).
5. Use the ‘Type’ filter to limit the type of articles returned by your search to one of the following options:
  - Research articles: Journal articles presenting the results of experimental research;
  - Reviews: Reviews on a topic covering a broad set of previously published articles;
  - Preprints: Research articles that have not yet undergone formal peer review and have been submitted to public preprint servers.
6. When selecting the ‘Preprints’ type filter it is possible to further limit your results using the ‘Journal published’ sub filter (Fig. 16). This will return preprints that are linked to a subsequent peer-reviewed journal publication.
7. Use the ‘Free full text’ filter to limit your search to ‘Free to read’ or ‘Free to read and use’ articles available in Europe PMC.
  Free full text is available from legal sources for many articles in Europe PMC. Articles with free full text can either be free to read or free to read and use, based on their licensing.
  - ‘Free to read’ filter includes articles that have free full text openly available in Europe PMC.
  - ‘Free to read and use’ filter includes articles that are made available under a Creative Commons license, or similar license. These articles are a part of the Europe PMC open access subset and can be reused, for example, to adapt a figure for a different publication, translate the original publication into another language, or include part of the publication in a textbook (Europe PMC, 2022d).
8. Use the ‘Date’ filter to limit your search to articles that were published within a specific year or a year range. You can select one or multiple boxes for the three most recent publication years. Alternatively, select the ‘Custom date range’ option, input publication years you are interested in, and use the search icon to search within the specific range (Fig. 16).
9. In addition to using search filters, search can be refined by combining search terms with Boolean operators, ***AND***, ***OR***, ***NOT***. For example, ***microvesicles AND exercise***, ***pathogens OR bacteria***, ***sepsis NOT covid*** (Fig. 17).
  Boolean operators are a logical connection between search terms. You can use the Boolean operators AND, OR, NOT to control search results in Europe PMC. Note that AND is a default Boolean operator used by the Europe PMC search to connect individual terms in a keyword string. For example, in Europe PMC a search for **metastatic bone cancer** is equivalent to a search for **metastatic AND bone AND cancer**.

## Finding Research By Author (Basic Protocol 6)

This protocol describes how the user can search for research publications and preprints by a particular author using Europe PMC.

### Necessary Resources

#### Hardware

Device capable of supporting a Web browser and an Internet connection

#### Software

Up-to-date Web browser (e.g., Firefox, Apple Safari, Google Chrome)

**Figure 18:**
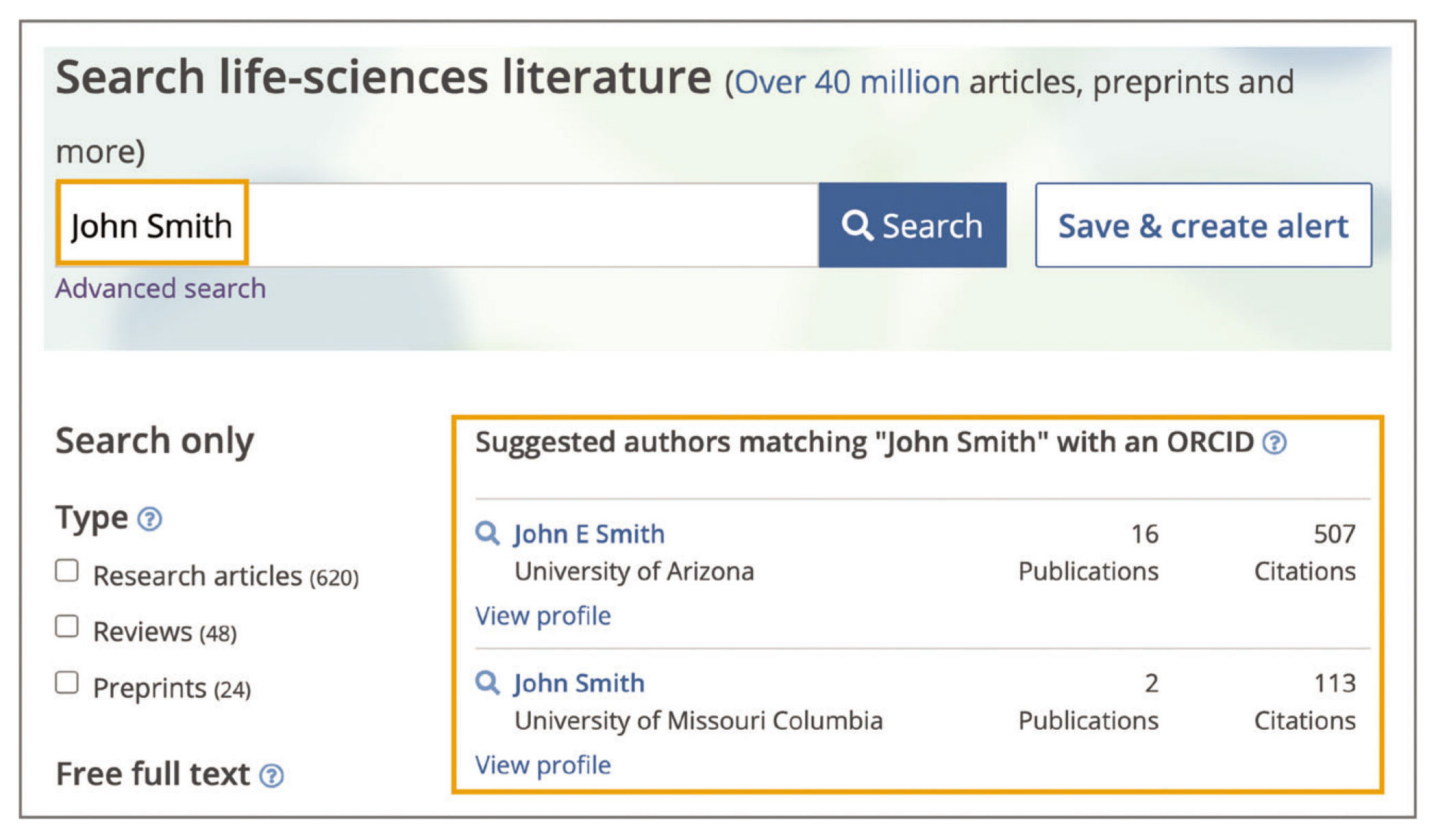
Search results for a ***John Smith*** author search. Two suggested authors matching John Smith with an ORCID are displayed at the top, along with the number of publications and citations in Europe PMC. URL used for the screenshot: https://europepmc.org/search?query=John%20Smith

1. Type author’s last name and first name or initial(s), for example ***John Smith*** or ***Smith J***, into the Europe PMC main search bar. Up to two authors with a matching name will be displayed at the top of the search results in the ‘Suggested authors’ box as shown in Figure 18.
  Searching for **John Smith** would return publications from authors named John Smith, whereas searching for **Smith J** would return publications from authors with a first name beginning with J and last name Smith, for example John Smith or James Smith.
  Suggested authors feature is only available for author matches where authors have a public ORCID profile and at least one publication from their ORCID profile is available in Europe PMC. ORCID stands for Open Researcher and Contributor Identifier; it is a unique identification number to distinguish individual research authors (ORCID, 2022). Suggested authors are ordered by the highest total number of publications they have in Europe PMC.
2. To limit your search to one of the suggested authors, use the hyperlinked author name in the ‘Suggested authors’ box. This will initiate an ORCID author search. It is also possible to search directly for a specific ORCID ID. To search for an author by ORCID, type the ORCID ID, such as ***0000-0002-1611-6935***, in the main search bar.
  Searching by ORCID allows users to refine search results for authors with similar names, limiting the results to articles by the author the ORCID is linked to only. When searching by ORCID in Europe PMC, only publications in Europe PMC in the author ORCID profile, and marked as visible to Public, Everyone, or Trusted Parties are displayed.

## Finding a Specific Article (Basic Protocol 7)

This protocol provides the user complete instructions on how to find a specific article based on bibliographic details, for example, title, journal, author, publication year.

### Necessary Resources

#### Hardware

Device capable of supporting a Web browser and an Internet connection

#### Software

Up-to-date Web browser (e.g., Firefox, Apple Safari, Google Chrome)

**Figure 19:**
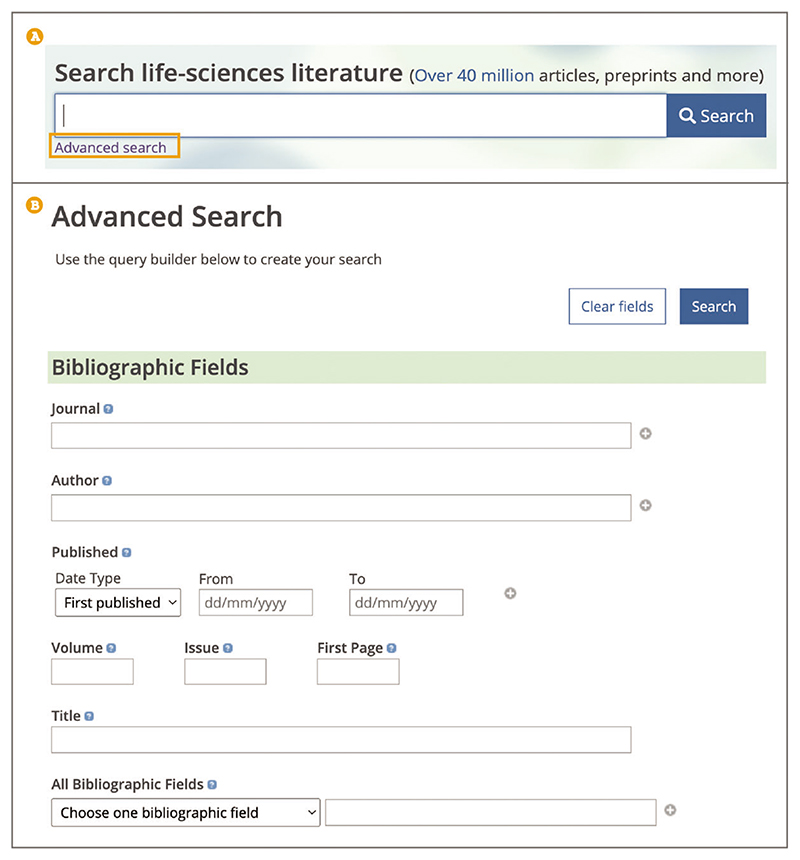
(**A**) The Advanced search link is highlighted under the main search bar. (**B**) Advanced search bibliographic fields query builder that enables the user to create their search.

1. To find an article by title, copy and paste the title as your keywords into the search bar, for example Protecting the Mitochondria in Cardiac Disease.
  For this to be effective the sort order needs to be set to the default option, relevance.
2. To find a specific article it may be simpler to use the Europe PMC Advanced search, especially if using a combination of bibliographic details, such as title, journal, author, and/or publication date, as it allows you to search in specific fields without needing to know the search syntax. Europe PMC Advanced search is a more targeted search; it is used to refine a search to return more relevant articles. To open Advanced search, use the hyperlink below the main search bar (Fig. 19A). Use the ‘Bibliographic Fields’ section at the top of the Advanced search page to construct your search. For example, if you know that the article title contains ***breast cancer***, it has been published by an author named ***Easton*** in ***The New England Journal of Medicine***, and the publication date is ***2021***, you can input these details into corresponding bibliographic search fields of the Advanced search (Fig. 19B). Once you have added all bibliographic details, use the ‘Search’ button at the top of the Advanced search page.
  Journal and author names are suggested as you type. Enter one author name per text field or use the ‘+’button to add another ‘Author’field for multi author searches. Note that using the ‘Title’ field of the Advanced search results in an exact phrase search, as described in step 3, Basic Protocol 1.

## Finding Information About a Methodology (Basic Protocol 8)

This protocol describes how to use a section-specific search to find examples of applying a particular method, exploring the methodology used in the article in more detail, and accessing additional protocols and relevant resources for your own research.

### Necessary Resources

#### Hardware

Device capable of supporting a Web browser and an Internet connection

#### Software

Up-to-date Web browser (e.g., Firefox, Apple Safari, Google Chrome)

**Figure 20:**
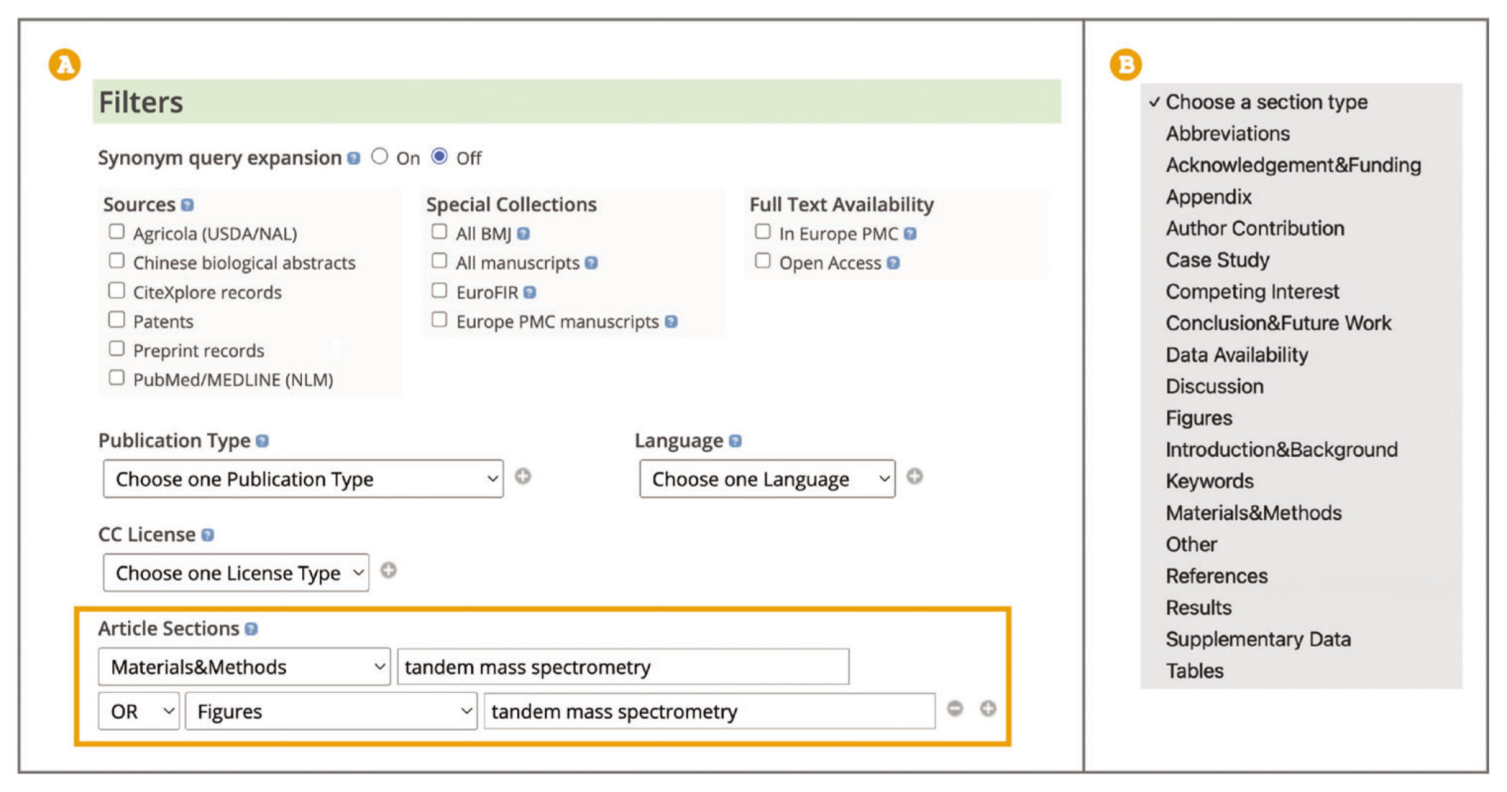
(**A**) Advanced search Filters section. Search terms for ***tandem mass spectrometry*** entered in the Article sections text box. Materials & Methods and Figures are selected from the drop-down menu and combined with a Boolean OR. (**B**) Article sections drop-down menu options.

**Figure 21:**
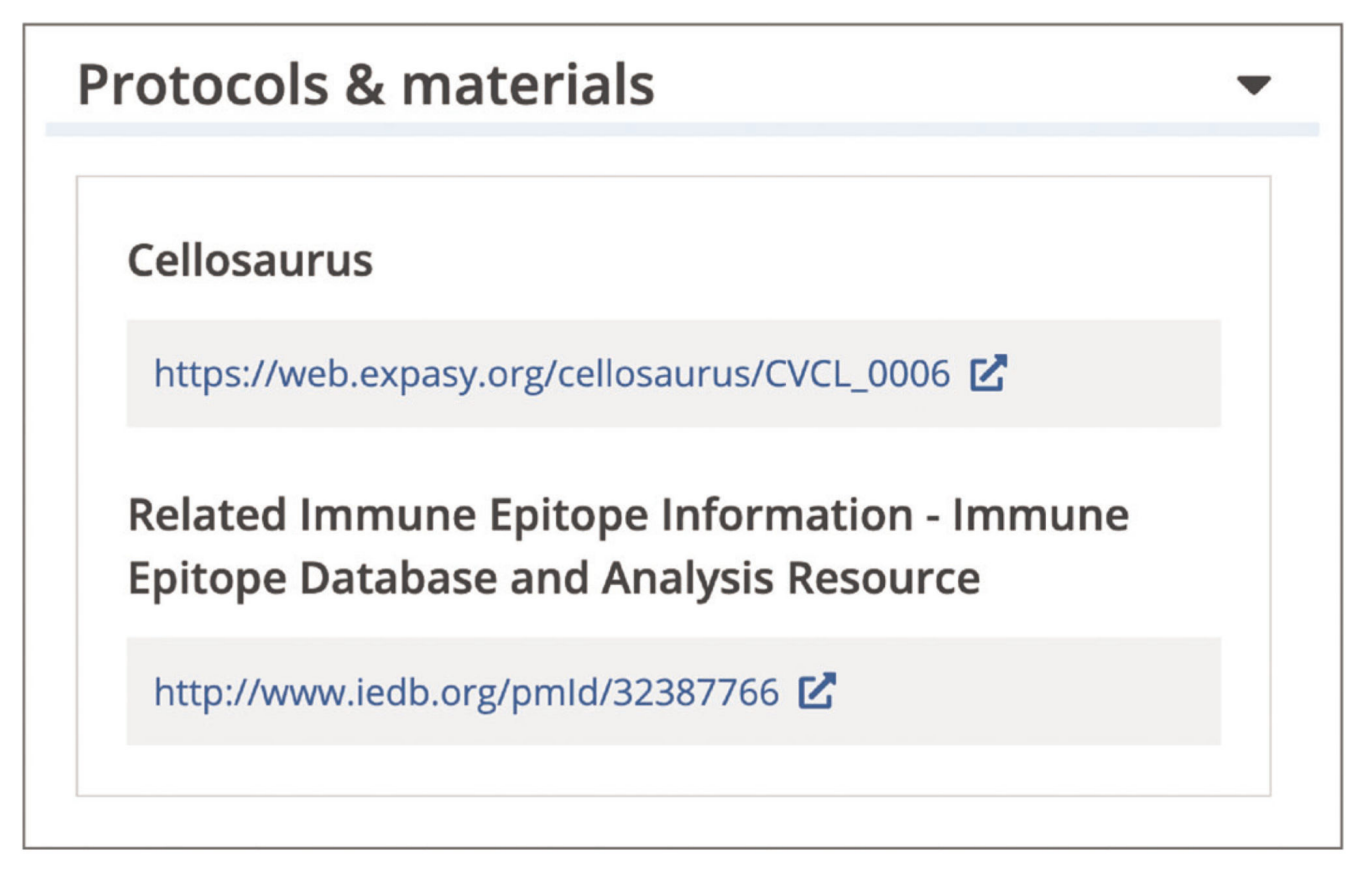
Protocols & materials section on the article page. Links to the relevant cell line in Cellosaurus (Expasy, 2022) and data on antibody and T-cell epitopes in the Immune Epitope Database (IEDB, 2022) are displayed. Article URL used for the screenshot: https://europepmc.org/article/MED/32387766

1. To search for a method by keyword or phrase, follow steps described in Basic Protocol 1.
2. To limit your search for a method of interest to a relevant article section, such as ‘Materials & Methods’, ‘Supplementary Data’, ‘Figures’, or ‘Tables’, use the Advanced search. When on the Advanced search page (see step 2, Basic Protocol 7) scroll down to the ‘Filters’ section (Fig. 20A). Under the ‘Article Sections’ heading use the ‘Choose a section type’ drop-down box to select a section of interest (Fig. 20B). Enter key terms or a phrase into the text box. It is possible to search across more than one section. Use the ‘+’ button to add another Section field. Scroll to the bottom of the Advanced search page and use the ‘Search’ button.
  Article section search is available only for full-text articles and preprints in Europe PMC. When searching through multiple sections, they are combined using Boolean AND (see step 8 of Basic Protocol 5 for more information about Boolean search). Alternatively, you can combine sections using OR or NOT by selecting an appropriate option (Fig. 20A).
3. To examine materials and methods used in a publication, open the article page and use the navigation bar to jump to the section of interest, such as ‘Materials & Methods’, in the full text of the article (see step 1, Basic Protocol 3 for guidance on using the navigation bar).
4. You can find additional information on protocols, reagents, cell lines, and other resources used or generated in the publication by opening the ‘Protocols & materials’ section in the left navigation bar on the article page (Fig. 21).
  The Protocols & Materials section is generated by Europe PMC based on links provided by external services, such as protocols.io (protocols.io, 2022), Ximbio (Ximbio, 2022), Cellosaurus (Expasy, 2022), and others. This section will only appear within the article if links to additional resources have been provided by those external services for that article, which are then stored in the Europe PMC database. Currently, this is only available for a limited set of articles because it is a new innovative feature to support researchers, which Europe PMC is planning to expand further in the years to come. If no link is established, you can find information on the protocols used in the research paper full text and follow any relevant references.
5. To identify experimental methods, cell lines, chemicals, clinical drugs, or organisms in the article text, open the ‘Annotations’ panel and select terms of interest (described in steps 3-4, Basic Protocol 3).

## Finding Evidence of Biological Interactions, Relations, and Modifications (Basic Protocol 9)

This protocol explains how to find current published research presenting scientific evidence, such as gene-disease relations or protein interactions, to support and extend your research, for example if the gene you are researching is involved in any diseases. Here we describe the steps to identify publications mentioning biological events, including interactions, relations, and modifications, as well as ways to access further data on the interaction of interest.

### Necessary Resources

#### Hardware

Device capable of supporting a Web browser and an Internet connection

#### Software

Up-to-date Web browser (e.g., Firefox, Apple Safari, Google Chrome)

**Figure 22:**
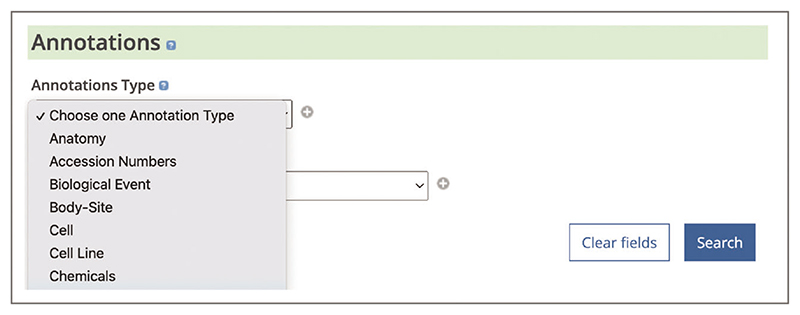
Annotations section on the Advanced search page with the drop-down box showing some of the Annotation types available to select.

**Figure 23:**
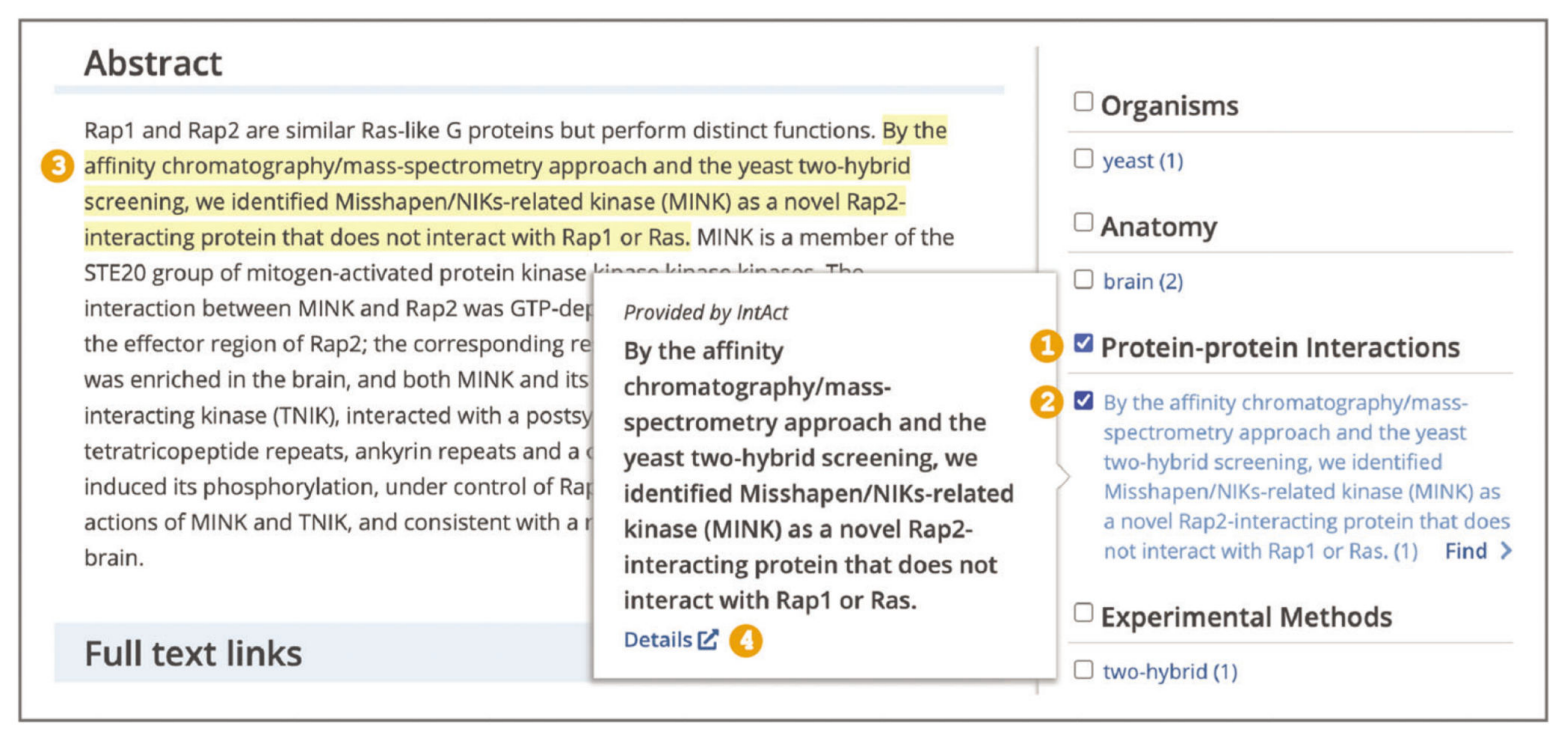
Article page view with the Annotations panel open. Protein-protein Interactions category is selected (1), with the MINK Rap-2 interaction annotation selected (2) and highlighted in the text (3), the pop-up window displays the link to the IntAct database record for this interaction (4). Article URL used for the screenshot: https://europepmc.org/article/MED/18930710

1. To find publications reporting evidence of biological interactions, relations, and modifications use the Advanced search. When on the Advanced search page (see step 2, Basic Protocol 7) scroll down to the ‘Annotations’ section (Fig. 22). Under the ‘Annotations Type’ heading use the ‘Choose one Annotation Type’ drop-down box to select annotations of interest. Available annotation types include: Transcription factors–Gene targets, Protein–protein Interactions, Genetic mutations, Biological events (Phosphorylation), Gene–Disease associations, Gene Function, and others. Use the ‘+’ button to add more annotation types. Use the ‘Search’ button at the bottom of the Advanced search page.
2. To identify biological interactions, relations, and modifications in the article text, open the ‘Annotations’ panel and select concepts of interest (described in steps 3-4, Basic Protocol 3).
3. To access further evidence for the annotation of interest, hover over the highlighted concept in the text or in the ‘Annotations’ panel to open a pop-up window. The pop-up window displays a link to the related database record (Fig. 23). For example, protein interaction annotations will be linked to a corresponding record in the IntAct database, providing interaction details, such as interaction type, detection method, or host organism (IntAct, 2022).

## Finding Data Behind a Publication (Basic Protocol 10)

Modern biological studies often generate several types of data that may reside in different places, creating a challenge to locate all the data pertaining to a study. This protocol explains how to access and examine the data relevant to the publication, find articles citing a particular data type, such as electron microscopy tomograms, or find articles referenced by a particular data resource, such as FlyBase (FlyBase, 2022).

### Necessary Resources

#### Hardware

Device capable of supporting a Web browser and an Internet connection

#### Software

Up-to-date Web browser (e.g., Firefox, Apple Safari, Google Chrome)

**Figure 24:**
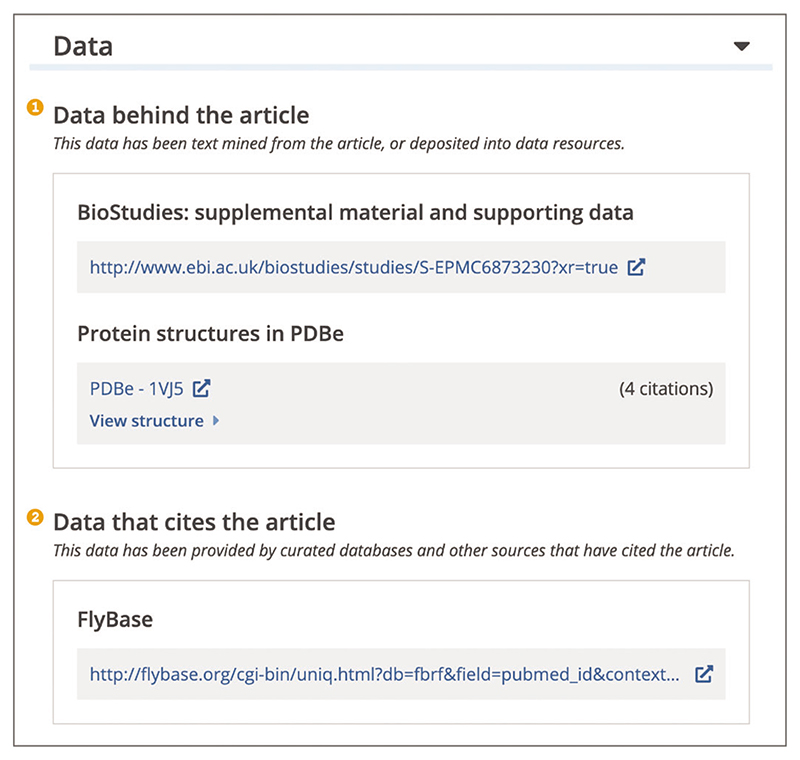
Data section on the article page. Data behind the article section (1) displaying a link to the BioStudies record for supplemental material and supporting data, as well as a PDBe-1VJ5 data citation for a protein structure in the PDBe database (EMBL-EBI, 2022a; PDBe, 2022). Data citing the article section (2) displaying a link to the FlyBase data record referencing the publication. Article URL used for the screenshot: https://europepmc.org/article/MED/30472438

**Figure 25:**
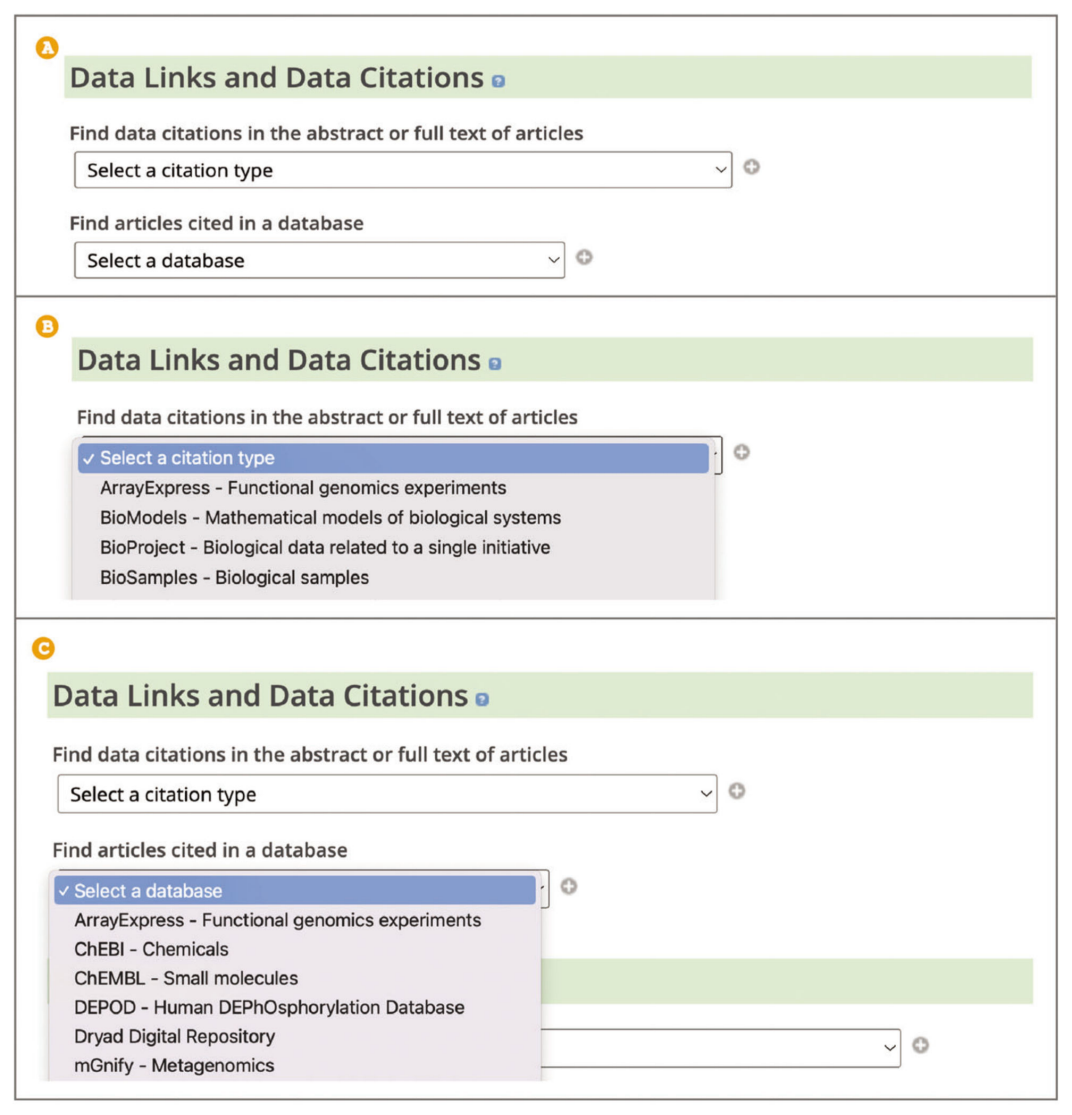
(**A**) View of the Data Links and Data Citations section of the Advanced search. (**B**) Find data citations in the abstract or full text of articles section search showing some drop-down options. (**C**) Find articles cited in a database section search showing some drop-down options.

1. To access the data relevant to the publication, open the article page and use the navigation bar to locate the ‘Data’ section. The ‘Data’ section encompasses links to supplemental and supporting data listed in the ‘Data behind the article’ section, and related or curated data listed in the ‘Data that cites the article’ section (Fig. 24). To access and examine the data, open the hyperlink to the data record in an external repository.
  The ‘Data behind the article’section contains the link to supplementary materials, as well as data accessions and data digital object identifiers (DOIs) cited in the article text and identified by text-mining. Data accessions are unique numbers assigned by databases as a means of identifying specific datasets. Cited data is linked to the corresponding record in the external database, for example 2p1l accession cited in the article text will be linked to the PDBe entry for the “Structure of the Bcl-XL:Beclin 1 complex”. The ‘Data citing the article’ section is based on links provided by public life science databases, such as FlyBase (FlyBase, 2022), UniProt (UniProt, 2022), ChEMBL (chEMBL, 2022), and others. When database records are submitted or curated, references to the literature are frequently added. For example, IntAct data record for MLLT11-TRIL protein interaction will cite the “MLLT11-TRIL complex promotes the progression of endometrial cancer through PI3K/AKT/mTOR signaling pathway” publication that reported this interaction (IntAct, 2022).
2. To find publications citing data from a specific resource, use the Advanced search. When on the Advanced search page (see step 2, Basic Protocol 7) scroll down to the ‘Data Links and Data Citations’ section (Fig. 25).
3. To find articles citing a particular data type, such as functional genomics experiments or non-coding RNA sequences, under the ‘Find data citations in the abstract or full text of articles’ heading use the ‘Select a citation type’ drop-down box to select the data citation of interest (Fig. 25). Use the ‘+’ button to add more data types. Use the ‘Search’ button at the bottom of the Advanced search page.
4. To find articles referenced by a particular data resource, such as FlyBase (FlyBase, 2022), UniProt (UniProt, 2022), or OMIM (OMIM, 2022), under the ‘Find articles cited in a database’ heading use the ‘Select a database’ drop-down box to select the database of interest (Fig. 25). Use the ‘+’ button to add more data types. Use the ‘Search’ button at the bottom of the Advanced search page.

## Expanding a Reading List and Building a Bibliography (Basic Protocol 11)

This protocol provides the user with multiple ways to expand their reading list by looking at references, citing, and similar articles, and explains how to add articles of interest to a citation manager to build and maintain a bibliography.

### Necessary Resources

#### Hardware

Device capable of supporting a Web browser and an Internet connection

#### Software

Up-to-date Web browser (e.g., Firefox, Apple Safari, Google Chrome)

**Figure 26:**
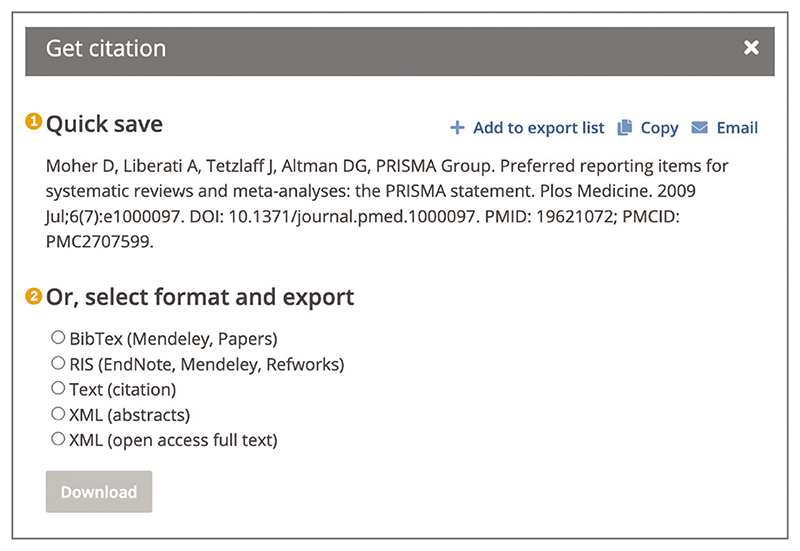
The Get citation pop up box for an article being viewed in Europe PMC. This provides the user with two options: Quick save (1), Or, select format and export (2). Article URL used for the screenshot: https://europepmc.org/article/MED/19621072

**Figure 27:**
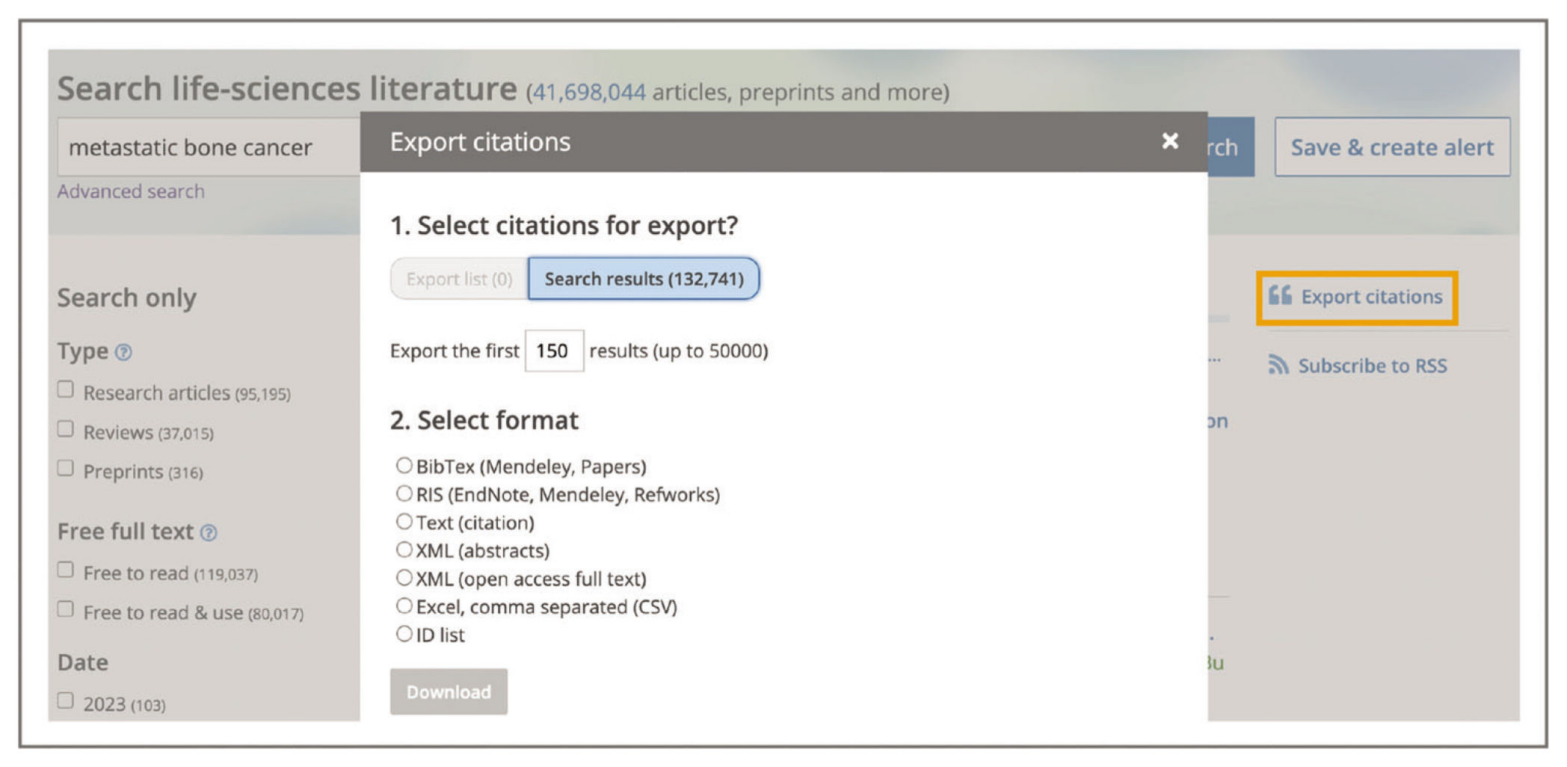
Search results for the terms ***metastatic bone cancer*** entered into the Europe PMC main search bar with pop up box to export citations open and the export citations button in the right-hand bar highlighted in a box. This method of citation exportation allows you to export up to 50,000 articles and choose the desired format.

1. To read publications cited by the article or preprint of interest, select the ‘Free full text’ section in the navigation bar to expand it and then open the ‘References’ section (Fig. 6A). To access an article from the reference list, open the ‘[Europe PMC free article]’ or ‘[Abstract]’ hyperlink for references that are available in Europe PMC. You can alternatively access the reference from the publisher website or via Google Scholar by selecting ‘[Crossref]’ or ‘[Google Scholar]’ hyperlink.
  Reference lists are available for publications that have full text in Europe PMC.
2. To expand your reading list with articles that cite the publication of interest, use the navigation bar to go to the ‘Citations & impact’ section (Fig. 6B). Scroll to ‘Article citations’ section, which will display the five most recently published articles citing the article you were reading. To view the full list of citing articles open the ‘Go to all [number of] article citations’ link at the bottom of the ‘Article citations’ section, which will take you to the Europe PMC search page displaying all citing articles.
3. To expand your reading list with similar articles, use the navigation bar to jump to the ‘Similar articles’ section (Fig. 6B). ‘Similar articles’ provides a list of the top five similar articles to the one you are reading.
  To derive the list of similar articles, Europe PMC uses a word-weighted algorithm to compare words from the title and abstract of each citation (Lin et al., 2007).
4. To add an article you are reading to a citation manager of choice, use the ‘Get citation’ option from the right hand toolbar. This will open a pop-up box with options to quickly save citation details or to export citation in a format of choice (Fig. 26). Format options include BibTex format (suitable for export to Mendeley and Papers), RIS format (suitable for export to EndNote, Mendeley, and Refworks), Text to download citation details in .txt format, and XML for abstracts or open access full-text articles, where available (EndNote, 2022; Mendeley, 2022; Papers, 2022; RefWorks, 2022).
  Quick save provides citation in the National Library of Medicine (NLM) format (NIH, 2018); this can be added to an export list, copied, or emailed using the icons in the top right corner (Fig. 26).
5. To add a list of publications to a citation manager of choice from the search results page listing publications of interest, select the ‘Export citations’ link on the left-hand side (Fig. 27A). This will open a pop-up box, where you can select citations for export and choose the desired format (Fig. 27B).
  You can export up to 50,000 of the first listed results.

## Staying on Top of the Current Literature (Basic Protocol 12)

This protocol walks the user through setting up an email alert to keep them up to date with the current literature in their fields of interest. Alerts can be set to stay on top of the new preprints, reviews, and journal articles on a particular topic, to follow an author, research group, or an institution, or to track journals or preprint servers covering a particular research field.

### Necessary Resources

#### Hardware

Device capable of supporting a Web browser and an Internet connection

#### Software

Up-to-date Web browser (e.g., Firefox, Apple Safari, Google Chrome)

**Figure 28:**
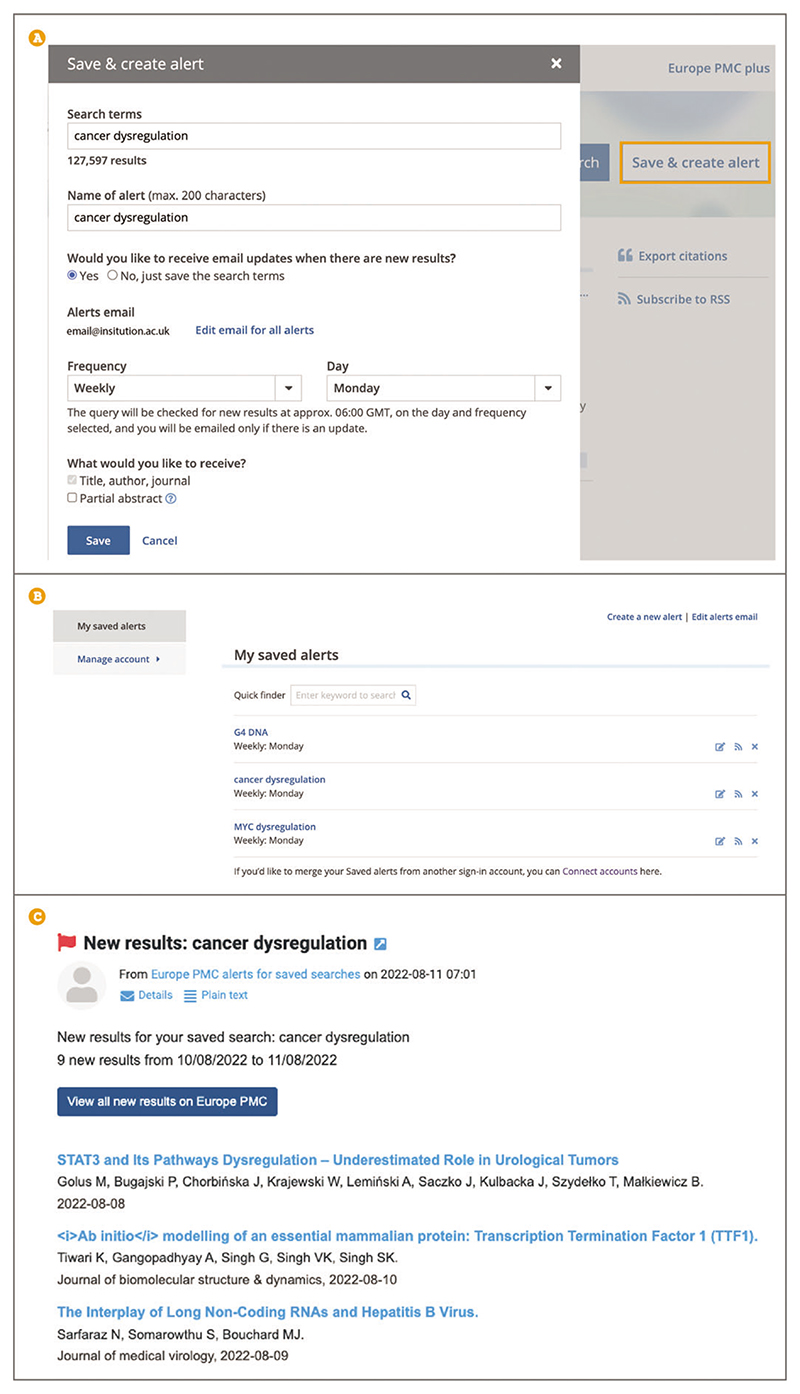
(**A**) Search page with a Save & create alert pop up box populated for the search terms ***cancer dysregulation*** to receive weekly updates every Monday to the email listed on the user’s account. (**B**) A user account with some saved alerts, including ***cancer dysregulation*** updates to be delivered weekly on a Monday. (**C**) A user’s email showing the alert arriving in their inbox for ***cancer dysregulation*** search.

1. To stay on top of new published literature you can set up email alerts for new results of keyword searches. To set up an alert for your search of choice select ‘Save & create alert’ in the box beside the main search bar on the search results page (Fig. 28A).
  Terms can describe a research area of interest, for example **cancer dysregulation**, an author’s name, such as **Ayman Saleh**, a research institute, such as **John Innes Centre**, or preprint and/or journal servers, for example **bioRxiv**.
2. When creating an alert you will be asked to sign in with an ORCID (ORCID, 2022), Twitter (Twitter, 2022), or Europe PMC account. You can access, modify, or delete any of your saved alerts from your account (Fig. 28B).
  Having an account is required for the alert setup in order to provide an email address that Europe PMC has permission to send the alert results to.
3. Once you are signed into your account, you will be able to fill out the ‘Save & create alert’ form (Fig. 28A). The name of the alert will be automatically filled out to match your search terms, but you can edit the alert name to help you identify the alert easily. You can choose to receive regular email updates or to save the search to run yourself at any time. Saved searches can be accessed by logging into your user account. Email updates can be set up to be sent as soon as available, weekly, or monthly by choosing the desired option from the ‘Frequency’ drop down menu. For weekly and monthly alerts, you will need to select a preferred day to receive the new updates. Any new results are emailed at ~06:00 GMT. Finally, you can choose to receive the title, author, and journal for any new result, or opt for the partial abstract option, which will include the first few lines of the abstract in the email update (Fig. 28C).

## Commentary

### Background Information

Europe PMC is an open access database that provides comprehensive access to life sciences literature from trusted sources. This database is a service supported by the Europe PMC Funders’ group, developed by the European Bioinformatics Institute (EBI; EMBL-EBI, 2022b); and in cooperation with the National Center for Biotechnology Information at the U.S. National Library of Medicine (NCBI/NLM; NCBI/NLM, 2022). Europe PMC is a part of the ELIXIR infrastructure, and an ELIXIR Core Data Resource (ELIXIR, 2022). Europe PMC partners with other organizations to build robust public tools to provide open content and data, and to advance life sciences research. The database includes over 41 million publications, preprints, and other documents enriched with links to supporting data, reviews, protocols, and other relevant resources. The protocols in this publication walk the user through how to effectively search and evaluate the content in Europe PMC.

### Critical Parameters

When choosing the key term(s) for your search, it is particularly important to consider which terms are relevant to your search, which term(s) are frequently used in publications in your field, and what synonyms and alternative spellings exist for your search terms, in order to compose an effective and relevant search string. Make sure you use appropriate connections for your search terms. The Boolean operator AND is used by default, however, you can also use the Boolean operators OR and NOT to tailor your search, as discussed in Basic Protocol 5 and shown in Figure 17. Sort order determines which search results are shown first; make sure an appropriate sort order is selected. Default sort order is set to Relevance; it can also be changed to Most recently added, Times cited, and Date published as discussed in Basic Protocol 5. Users should select appropriate search filters, as this will limit search results. Available filters include Type, Free full text, and Date as discussed in Basic Protocol 5.

Where key terms are found in the publication, text can affect the relevance of search results. For example, the same keyword might refer to an author, journal, or biological concept. To compose a more efficient search you should consider using the Advanced search, where you can search for key term(s) in specific article sections (Basic Protocol 8), or bibliographic fields (Basic Protocol 7).

### Troubleshooting

See Table 1 for common problems encountered when performing these protocols and suggested solutions.

### Understanding Results

Europe PMC results are literature records including all the abstracts in PubMed and some other large sets of abstracts not available through PubMed such as Agricola (AGRICOLA, 2022; National Library of Medicine, 2022). A record entry will contain a title, authors, journal name, date of publication, unique identifiers such as a DOI, abstract, and the full text if available. On the article page all these will be displayed whereas on the search results page the abstract and full text will not be displayed. Note that snippets are also displayed on the search results page for key term(s) searched for in the main search bar; for more information about snippets see Basic Protocol 5.

### Time Considerations

Due to the nature of searching the literature and the common practice of iterating searches over and over to refine or expand results, specific timings cannot be provided.

## Figures and Tables

**Table 1:** Sources and Solutions to Potential Errors

Problem	Possible Cause	Solution
Too many records returned for a search	The search terms were too broad or generic	To limit the number of search results: Where possible, replace general search terms with more specific ones (e.g., search for ***breast cancer*** instead of ***cancer)*** Include additional terms in your search query Use filters to restrict by article type or publication date Use the Boolean operator NOT to remove results containing terms that are not relevant to your search Use Advanced search to limit search to specific fields, such as title, rather than searching anywhere in the full text Use quotation marks to find exact matches
None or fewer than expected records are returned for a search	The search was too specific or refined	To expand the search: Check spelling for typos and mistakes. If necessary, use the spelling option suggested by the Did you mean functionality. Where possible, consider removing filters or broadening your selection criteria If applicable, consider searching through the full text as opposed to specific sections, such as abstract Turn on synonyms in the Advanced search to search for records that contain terms synonymous to your search terms Use the Boolean OR to add alternative spellings to your search Use the wildcard asterisk to search for word variations
Exact phrase search returns no results	The exact phrase terms are not adjacent in any records	Run the search without quotation marks with the Sort by set to relevance
Advanced search returns no results	Advanced search adds quotation marks to all field searches, resulting in an exact match, for example TITLE: “diabetes mellitus”	Search for multiple terms in the same field, such as TITLE, by adding Advanced search field options with a plus sign. Input a single search term or a search phrase per search box.
Displayed results are not relevant	The sort order is not set to relevance A phrase is searched without using double quotes Search terms are found in the full text, but not in the section of interest, e.g., abstract or title	Use search snippets to assess the relevance of your search result If the sort order was changed from its default setting, change it back to relevance When searching for a phrase, such as ***cell* cycle**, make sure to use double quotes. Otherwise, results containing both search terms not adjacent to each other, for example …circadian cycle in the cell…, may be returned. In some cases, search terms are mentioned in an article section that is not relevant to the search, for example in a table or supplemental files, but not in the abstract. You can restrict your search to the desired article section, such as Results, by using the Advanced search.
Author search returns publications from another author	For authors with common names, such as Wang Cong, a search by name and surname returns publications by other researchers	Search using the author’s ORCID, an identifier unique to that specific author. Paste the ORCID, such as 0000-0002-1611-6935, into the main search bar. Look up an ORCID using the Author field of the Advanced search. Start typing the author’s name and select relevant author ORCID from the autosuggest drop-down list.
Full text is missing	The full text is not included in Europe PMC	Free full text available from external sources can be accessed on the article page from the Full text section displayed in the navigation bar. Alternatively, use the Free full text filter to limit your search for articles with full text available in Europe PMC.
Navigation headings are missing on the article page	Navigation headings are only available if the article full text is included in Europe PMC, or if the additional information, such as reviews or protocols, is available from external providers	To return full-text articles, use the Free full text filter. To find articles with certain headings, such as Reviews, use the Advanced search and select the relevant review provider from the External links drop-down menu.

## Data Availability

Data sharing not applicable to this article as no datasets were generated or analyzed during the current study
